# Process conditions affect microbial diversity and activity in a haloalkaline biodesulfurization system

**DOI:** 10.1128/aem.01864-23

**Published:** 2023-12-11

**Authors:** Suyash Gupta, Rieks de Rink, Johannes B. M. Klok, Gerard Muyzer, Caroline M. Plugge

**Affiliations:** 1 Wetsus, European Centre of Excellence for Sustainable Water Technology, Leeuwarden, the Netherlands; 2 Microbial Systems Ecology, Department of Freshwater and Marine Ecology, Institute for Biodiversity and Ecosystem Dynamics, University of Amsterdam, Amsterdam, the Netherlands; 3 Environmental Technology, Wageningen University & Research, Wageningen, the Netherlands; 4 Paqell B.V., Utrecht, the Netherlands; 5 Laboratory of Microbiology, Wageningen University & Research, Wageningen, the Netherlands; Kyoto University, Kyoto, Japan

**Keywords:** active microbes, sulfide oxidation, sulfur-oxidizing bacteria (SOB), 16S rRNA, cDNA, *Thioalkalivibrio*, *Alkalilimnicola*, Thiopaq

## Abstract

Biodesulfurization (BD) systems that treat sour gas employ mixtures of haloalkaliphilic sulfur-oxidizing bacteria to convert sulfide to elemental sulfur. In the past years, these systems have seen major technical innovations that have led to changes in microbial community composition. Different studies have identified and discussed the microbial communities in both traditional and improved systems. However, these studies do not identify metabolically active community members and merely focus on members’ presence/absence. Therefore, their results cannot confirm the activity and role of certain bacteria in the BD system. To investigate the active community members, we determined the microbial communities of six different runs of a pilot-scale BD system. 16S rRNA gene-based amplicon sequencing was performed using both DNA and RNA. A comparison of the DNA- and RNA-based sequencing results identified the active microbes in the BD system. Statistical analyses indicated that not all the existing microbes were actively involved in the system and that microbial communities continuously evolved during the operation. At the end of the run, strains affiliated with *Alkalilimnicola ehrlichii* and *Thioalkalivibrio sulfidiphilus* were confirmed as the most active key bacteria in the BD system. This study determined that microbial communities were shaped predominantly by the combination of hydraulic retention time (HRT) and sulfide concentration in the anoxic reactor and, to a lesser extent, by other operational parameters.

**IMPORTANCE:**

Haloalkaliphilic sulfur-oxidizing bacteria are integral to biodesulfurization (BD) systems and are responsible for converting sulfide to sulfur. To understand the cause of conversions occurring in the BD systems, knowing which bacteria are present and active in the systems is essential. So far, only a few studies have investigated the BD system’s microbial composition, but none have identified the active microbial community. Here, we reveal the metabolically active community, their succession, and their influence on product formation.

## INTRODUCTION

Hydrogen sulfide (H_2_S) is a malodorous and toxic environmental pollutant that must be removed from industrial gas streams before its release into the atmosphere. There are several options for removing H_2_S from the source streams, but only one, the haloalkaline biodesulfurization (BD) process, is an eco-friendly and cost-effective technology. The process uses haloalkaliphilic sulfide-oxidizing bacteria (SOB) that oxidize H_2_S to elemental sulfur, which subsequently can be used in the production of fertilizers and fungicides (1–4). A traditional BD system consists of an absorber, an oxic bioreactor, and a sulfur recovery section. In the absorber column, H_2_S-rich gas is absorbed in the haloalkaline process solution as soluble bisulfide (HS^-^) via a countercurrent contact. The sulfide-rich process solution is then directed to an oxic bioreactor, where the dissolved sulfide species are oxidized by SOB into elemental sulfur (S_8_). The formed sulfur then goes to a sulfur recovery section where the sulfur is recovered (2, 3, 5).

The traditional BD process (“Thiopaq” process) is constantly studied to reduce the formation of unwanted by-products, i.e., sulfate and thiosulfate, by finding better ways to control the supply of oxygen (6) or by alternating the sulfidic and microaerophilic zone and consequently increasing the sulfur recovery (5). For the latter, a new process lineup with an additional bioreactor was designed, the “Thiopaq Ultra” process, and studied at the pilot scale (5). The lineup has, apart from an absorber and oxic bioreactor, an integrated anoxic bioreactor. Hence, by circulating the process solution through the setup, the reactors’ biomass, which includes SOB and other bacteria, gets exposed to alternating process conditions (2, 4, 5). With this new technology, the by-product (i.e., sulfate and thiosulfate) selectivity dropped from 22% to 3%, and caustic consumption and bleed formation decreased by 80%–90%, thereby increasing the recovery of elemental sulfur considerably (5, 7). These results make the Thiopaq Ultra technology more sustainable and cost-effective for desulfurizing sour gas streams from various industries.

In line with the product selectivity changes, the microbial composition of the system also changed and was mainly dominated by *Alkalilimnicola ehrlichii,* followed by *Thioalkalivibrio sulfidiphilus* (5, 7). A comparative microbial composition analysis showed that, in general, BD systems share a core microbial community (8). This core community comprises diverse microorganisms that may or may not be involved in sulfur conversions and have diverse metabolisms, such as auto- and heterotrophic (8). *Thioalkalivibrio sulfidiphilus* is an autotroph and a well-known sulfide oxidizer (9, 10). It was found to be the most abundant bacterial species in traditional BD systems (8–10). The other bacterium that became dominant in the new lineup was *Alkalilimnicola ehrlichii* (5, 7, 8, 11). It is a facultative chemoautotroph and facultative anaerobe that can oxidize sulfide to sulfur (12, 13). Some other abundant bacteria were affiliated with the genera *Halothiobacillus* (currently known as *Guyparkeria*)*, Thiomicrospira,* and *Halomonas* (8, 9, 14–20).

Apart from the technical change, studies have deduced the influence of the feed gas composition on the microbial community, e.g., the presence of toxic contaminants such as thiols and BTEX (8, 21, 22). Likewise, increasing the concentration of sulfide in the anoxic reactor and its hydraulic retention time (HRT) can impose an extra selection pressure on the microbial community (7).

The drawback of all the previous studies that show the microbial composition of the BD system is that they discuss only the presence of groups of bacteria at the end of the operation. They do not give information about the metabolically active members among the microorganisms found in the BD system. Furthermore, it was seen that BD systems were always inhabited by heterotrophs such as *Halomonas* and *Acholeplasma* and bacteria that are not known to oxidize sulfide (6–8). We hypothesize that these bacteria are metabolically less active compared to other chemoautotrophic bacteria found in these systems.

Several studies have used ribosomal RNA to identify and distinguish metabolically active microbial cells from inactive or dormant microbial cells (23–26). In a microbial community, a higher concentration of rRNA is linked with a high protein synthesis potential and can also predict the potential future activity of community members (25, 27).

In the present study, we determined the microbial communities in six runs of a pilot-scale Thiopaq Ultra system operated at different HRT and sulfide concentration using both DNA and RNA. We identified the active community members based on the higher relative abundance of RNA-based 16S rRNA amplicons over DNA-based amplicons. The 16S rRNA-based microbial community composition was monitored to study their succession over time and to determine their influence on the process performance.

## RESULTS

### Biodesulfurization process operation description

The characteristics of six different experimental runs of a pilot-scale BD installation are listed in Table 1 (7). The product selectivity of the six experimental runs of the pilot-scale BD system resulted in a selectivity for sulfur formation of 92.0%–96.7%. The term selectivity is generally applied in chemical engineering to describe the mole fractions of products that are formed from a substrate. The highest selectivity for sulfur formation was observed for Run 3 (96.7%), and the lowest was observed for Run 1 (92.0%) (Table 2). The highest sulfide loading rate resulted in the highest biomass concentration of 62 mg N L^−1^ and the highest specific sulfide uptake in the anoxic reactor of 3.99 mg S mg N^−1^ (Table 2). The biomass concentration and sulfate production rate increased with the length of the operation. In contrast, the alkalinity decreased with the duration of operation (Table 2; Dataset S1). The Spearman correlation analyses statistically confirmed these trends. The raw data of all parameters of the different runs are described in Dataset S1. These results were more explicitly described and discussed by de Rink et al. (7).

**TABLE 1:** Characteristics of six different experimental runs of a pilot-scale BD installation^a^

	S-load (g-S day^−1^)	Volume anoxic reactor (L)	Theoretical sulfide concentration anoxic reactor (g L^⁻1^)	HRT anoxic reactor (min)	Duration of operation (days)	Aerated volume (%)	Average pH Anoxic reactor	Average pH Oxic reactor	Average redox Anoxic reactor (mV)	Average Conductivity (mS/cm)	Average alkalinity (M)
Run 1	65	2.2	0.2	10	30	78.1	7.6 ± 0.1	8.4 ± 0.1	−420 ± 3	51.8 ± 3.7	0.55 ± 0.10
Run 2	65	6.0	0.2	30	31	62	7.7 ± 0.1	8.4 ± 0.1	−421 ± 2	61.1 ± 2.1	0.67 ± 0.06
Run 3	125	1.7	0.5	10	28	80.9	7.7 ± 0.2	8.5 ± 0.1	−428 ± 4	49.0 ± 2.3	0.58 ± 0.08
Run 4	170	1.5	0.9	10	47	82	7.8 ± 0.1	8.7 ± 0.2	−441 ± 7	48.5 ± 3.9	0.61 ± 0.11
Run 5	65	4.3	0.2	20	29	68.3	7.7 ± 0.1	8.4 ± 0.1	−423 ± 3	49.1 ± 1.5	0.58 ± 0.06
Run 6	125	3.5	0.5	20	43	72.2	7.5 ± 0.1	8.5 ± 0.1	−431 ± 4	51.0 ± 3.2	0.63 ± 0.04

^a^ As described in a previous study (7).

**TABLE 2:** Product selectivity and other performance parameters of six different experimental runs of a pilot-scale BD installation^a^

	Selectivity S_8_ (%)	Selectivity SO_4_^2−^ (%)	Selectivity S_2_O_3_^2−^ (%)	Average biomass concentration (mg N L^−1^)	Sulfide conversion rate oxic reactor (gL^−1^ day^−1^)	Specific sulfide uptake anoxic reactor (mg S mg N^−1^)
Run 1	92.0 ± 8.7	7.5 ± 9.2	0.5 ± 1.8	45	5.7	1.18 ± 0.51
Run 2	94.3 ± 4.8	5.6 ± 5.5	0.1 ± 1.3	43	5.7	1.37 ± 0.55
Run 3	96.7 ± 4.2	3.0 ± 3.4	0.3 ± 3.2	51	10.5	2.27 ± 0.69
Run 4	95.5 ± 3.3	3.9 ± 3.2	0.6 ± 0.7	62	14.9	3.99 ± 0.82
Run 5	95.0 ± 5.0	5.0 ± 5.0	−0.1 ± 1.3	44	5.7	1.86 ± 0.52
Run 6	95.0 ± 3.2	4.7 ± 3.2	0.4 ± 1.1	48	10.5	1.25 ± 1.07

^a^ Obtained from previously described data (7).

### Microbial diversity analyses

During six runs of the pilot reactor, the microbial diversity was analyzed using 16S rRNA gene-based DNA- and cDNA-based amplicon sequencing (112 samples in total, see Table S1). Four sequencing projects resulted in 2,153,910, 2,852,684, 2,817,954, and 4,906,018 sequence reads, respectively (Table S2). After denoising and chimera removal, 1,569,195, 1,920,826, 1,544,039, and 2,773,744 sequences remained, resulting in 192, 183, 283, and 240 Amplicon Sequence Variants (ASVs), respectively. Table S3 shows the summary of identified ASVs for each project and sequence, along with the number of samples sequenced for each run. The frequencies of all the ASVs obtained are shown in Tables S4 to S7. The ASV tables of the four sequencing projects were merged, resulting in 522 unique ASVs from 112 samples (Table S8). After removing ASVs with counts less than 2,000 in the merged table, we obtained 61 ASVs that represent the most prevalent sequences and were used for further analyses (Table S9).

**Fig 1:**
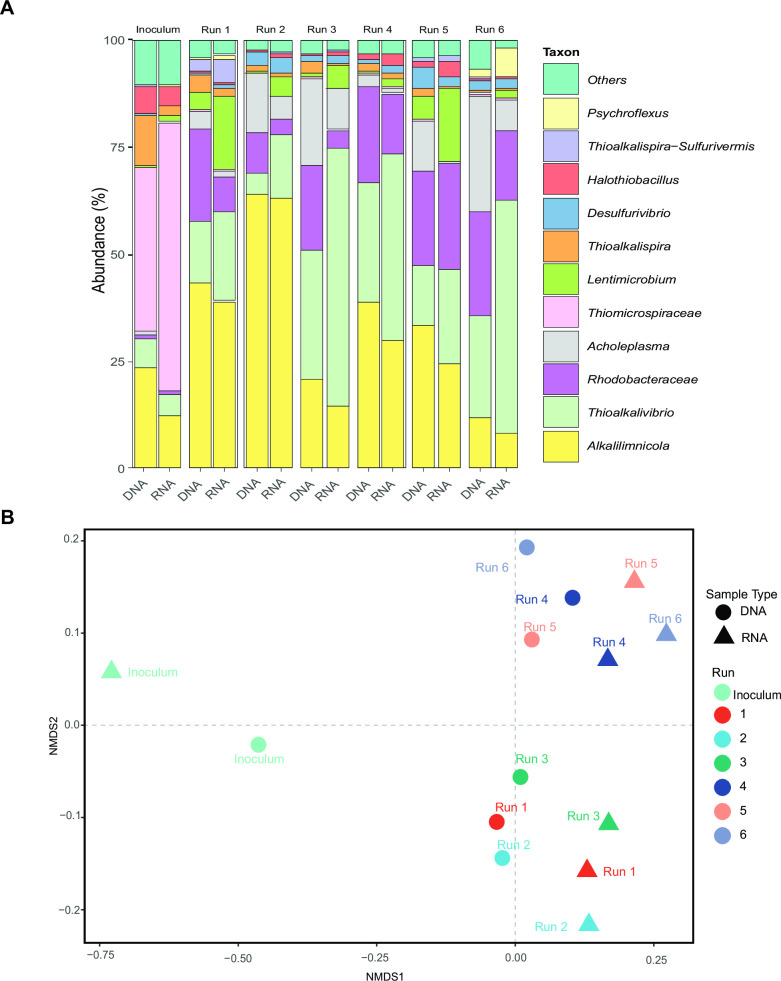
(**A**) Overview of the top 11 total (DNA) and active (RNA) genera and genus-like taxa in the inoculum and in the oxic reactor of the six runs at the end of operations. (**B**) Non-metric multidimensional scaling (NMDS) ordination plot of Bray–Curtis community dissimilarities based on ASVs of the 16S rRNA gene sequences from the inoculum and the oxic reactor of the six runs at the end of the operations.

Alpha- and beta-diversity analysis was performed to understand the effect of experimental runs (runs), HRT of anoxic reactor (HRT), sulfide concentration in anoxic reactor (sulfide concentration) and DNA/RNA based sample (sample type). The alpha diversity estimations denoting the number of ASVs indicated that diversity varied among the experimental runs (Table 3). The Kruskal-Wallis test showed that the number of ASVs was not significantly different in the three sections of the system. However, the number of ASVs significantly differed among runs with varying HRT and sulfide concentrations in the anoxic reactors (Table 3). The Shannon diversity was higher for HRTs and runs but not for the sulfide concentration (Table 3). The Shannon index (*H*) is a way to measure species diversity in a community that also takes species evenness into account (28, 29). The beta diversity estimations indicated that the microbial community composition differs among the runs (Table 3). The microbial composition also differs significantly based on the HRT, sulfide concentration, and sample type but not among the sections (Table 3). According to the Bray-Curtis dissimilarity estimations, the changes in the microbial composition could be explained in decreasing order by the parameters runs, HRT, sample type (DNA/RNA), and sulfide concentration. The UniFrac phylogenetic distance-based diversity index indicated a more substantial effect by the sample type and runs and a minor effect by the HRT and sulfide concentration (Table 3). The taxonomic bar plot (Fig. 1A) and the non-metric multidimensional scaling (NMDS) ordination plot using Bray-Curtis distances (Fig. 1B) clearly showed that the microbial composition of the inoculum that was used for all runs differed from the experimental runs’ composition.

**TABLE 3:** Summarized statistical test results for alpha and beta diversity measurements grouped by run, reactor section, sample type (DNA/RNA), and HRT and sulfide load in the anoxic reactor^*d*^

Factors	Alpha diversity significance (Kruskal-Wallis test)				Beta diversity significance (Adonis)						Beta diversity significance (Permanova)			
	Shannon		Observed features		Bray-Curtis dissimilarities			Weighted UniFrac dissimilarities			Bray-Curtis dissimilarities		Weighted UniFrac dissimilarities	
	*H^a^*	*P* value	*H*	*P* value	Pseudo *F^b^*	*R^c^*	*P* value	Pseudo *F*	*R*	*P* value	Pseudo *F*	*P* value	Pseudo *F*	*P* value
Runs	43.22	3.34e-8	42.43	4.82e-8	46.2	0.221	0.001	21.75	0.127	0.001	19.819	9.99e-05	11.761	9.99e-05
Sections	4.27	0.118	5.97	0.050	1.17	0.011	0.301	1.28	0.015	0.243	0.878	0.505	0.990	0.422
Sample type	3.36	0.066	10.54	0.001	21.59	0.103	0.001	24.37	0.143	0.001	13.537	9.99e-05	17.498	9.99e-05
HRT	27.28	1.1e-6	25.68	2.61e-6	24.67	0.118	0.001	4.91	0.029	0.002	21.888	9.99e-05	12.073	9.99e-05
Sulfide concentration	2.03	0.362	13.57	0.001	12.66	0.061	0.001	16.22	0.095	0.001	8.982	5e-04	8.811	9.99e-05

^*a*^ *H* is the Kruskal-Wallis test statistic.

^*b*^ Pseudo *F* test statistic is a ratio of the between-cluster variation to the within-cluster variation.

^*c*^ *R* is the coefficient of determination. It indicates the % change explained by a certain factor.

^*d*^ These tests do not include inoculum samples

### Active vs. total microbial community

The total and active community members in the haloalkaline BD system were determined by comparing 16S rRNA amplicons obtained from extracted DNA and RNA from the same sample (30). The sequences obtained from rDNA would indicate the total community present in the system, while the sequences obtained from rRNA would indicate the metabolically active community members. Figure 1A shows that members of the family *Thiomicrospiraceae,* including *Thioalkalimicrobium*, which is currently classified as *Thiomicrospira*, have a high relative abundance in the inoculum for both DNA and RNA. However, after around 30 days of operation, 16S rRNA of the genus *Thiomicrospira* became much less abundant for both DNA- and RNA-based estimations and even disappeared. At the end of the runs, the genera *Alkalilimnicola* and *Thioalkalivibrio* were the most abundant for both DNA- and RNA-based estimations compared to other community members. Minor and significant differences in both DNA- and RNA-based estimations were observed for other taxa. For instance, *Lentimicrobium* and *Thioalkalivibrio* had a higher rRNA abundance compared to rDNA. At the same time, other taxa like *Acholeplasma*, members of the family *Rhodobacteraceae,* and *Thiomicrospira* had higher rDNA abundance compared to rRNA. Figure 1B shows that for all runs and the inoculum, the active and total microbial communities varied from each other, which was also statistically confirmed (Table 3).

**Fig 2 F2:**
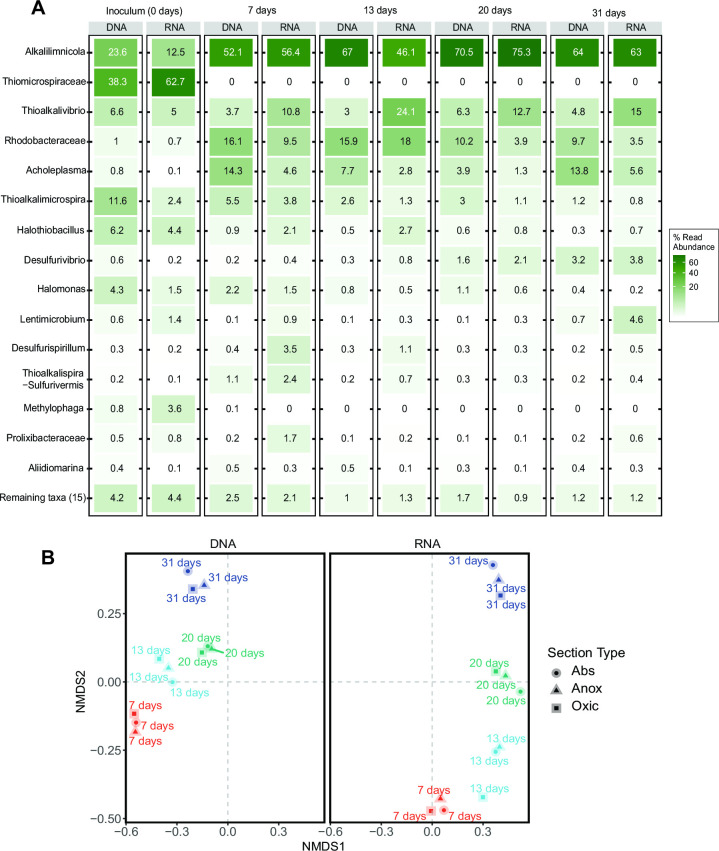
(**A**) Heatmap showing the relative abundance of the top 15 most abundant bacteria at the genus-like level for total (DNA) and active (RNA) communities for Run 2. (**B**) NMDS ordination plot of Bray-Curtis community dissimilarities among weekly DNA and RNA samples from all sections of the BD system showing changes in microbial composition over time for Run 2. The DNA and RNA samples were analyzed together but presented in two frames for better visualization.

Additionally, DNA- and RNA-based microbial compositions for Run 2 showed clear differences throughout the run (Fig. 2A and B). More results on Run 2 are shown in the next section.

As the differences between DNA-and RNA-based amplicon sequencing were significant and the microbial composition did not differ between the different process sections (i.e., absorber, anoxic reactor, and oxic reactor), all further analyses have been centered around RNA-based sequences from the oxic reactor in all runs. The core community analyses of RNA-based sequences from the oxic reactor resulted in a total of 27 ASVs that were present in all the samples. These 27 ASVs belonged to 20 genera listed in Table 4.

**TABLE 4:** List of core community members consistently present in the oxic section across all runs and time durations

Taxa	No. of ASVs
*Alkalilimnicola ehrlichii*	3
*Desulfurivibrio*	2
*Halomonas*	2
*Rhodobacteraceae*	2
*Thioalkalivibrio sulfidiphilus*	2
*Acholeplasma*	1
*Aliidiomarina*	1
*Desulfurispirillum indicum*	1
*Desulfuromusa*	1
*Ectothiorhodospiraceae*	1
*Halothiobacillus*	1
*Izimaplasma*	1
*Lentimicrobium*	1
*Nitrincola*	1
*Prolixibacteraceae*	1
*Psychroflexus*	1
*Thioalkalibacter*	1
*Thiomicrospira*	1
*Thioalkalispira-Sulfurivermis*	1
*Thioalkalivibrio halophilus*	1
*uncultured_Bacteroidetes/Chlorobi*	1

### Succession of the microbial communities

To get more insight into the succession of the microbial communities, we analyzed the operational run with the highest HRT in detail, i.e., Run 2, with an HRT of 30 min and a sulfide concentration of 0.2 gL^−1^ in the anoxic reactor (Table 1). The total and the active communities from all sections of the BD system were analyzed every week. After the first week, the community composition changed considerably in both abundance and activity (Fig. 2A and B). The taxonomic analysis of the microbial community showed that members of the genus *Alkalilimnicola* were most abundant and active throughout the whole run (Fig. 2A). Members of the genus *Thioalkalivibrio* were more active as compared to their abundance. Members of the family *Rhodobacteraceae* and the genera *Thiomicrospira* and *Acholeplasma* were more abundant than active (Fig. 2B).

### Effect of process parameters on the active community

**Fig 3:**
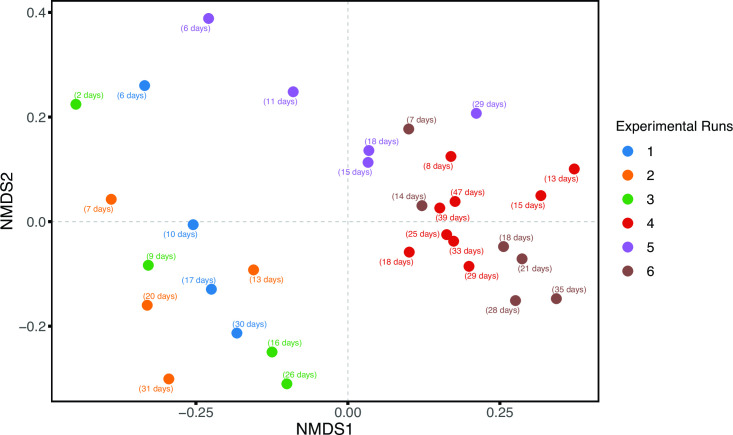
NMDS ordination plot of Bray-Curtis community dissimilarities for the active microbial communities in the oxic bioreactor of all six runs over time. The active fraction is analyzed using the 16S rRNA-based RNA amplicon sequencing (cDNA). For details about the runs see Table 1.

The distance-based NMDS plot (Fig. 3) helps us to visualize all the RNA samples (active fraction) together. Figure 3 clearly distinguishes between the highest HRT (30 min) and a sulfide concentration of 0.9 g/L but not for other runs. Statistics on the beta diversity of the active communities showed a significant effect of both HRT and sulfide concentration as an individual and combined effect (with *P* value = 0.005, *F* = 4,69, *R*^2^ = 0.11, and DF residuals = 28) (Fig. 3).

**Fig 4:**
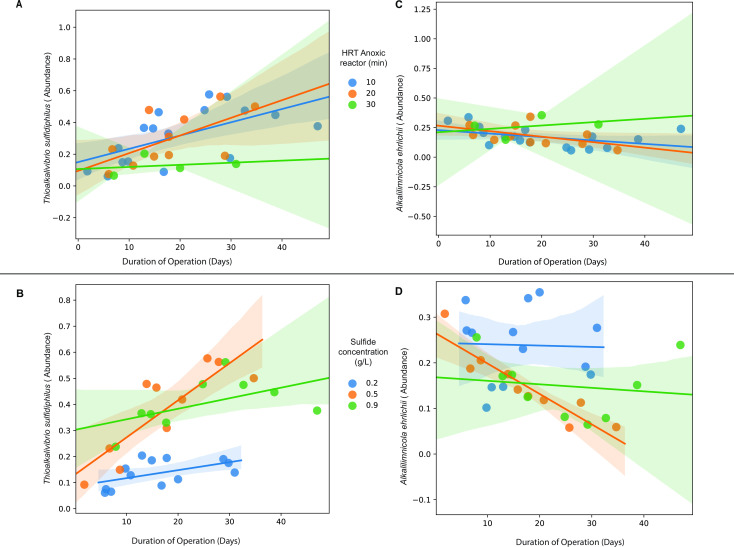
Regression scatterplots showing the predicted changes in the relative abundance of ASV “*65496bbf4dc78382fac58935a32b1445”* annotated as *Thioalkalivibrio sulfidiphilus* and ASV “*986967fd4264ba896aabed4c25f9a4af”* annotated as *Alkalilimnicola ehrlichii* over the duration of operation determined with a linear mixed model. The figures show the changes in the abundance of *Thioalkalivibrio sulfidiphilus* over time for (**A**) HRT of the anoxic reactor and (**B**) sulfide concentration. Similarly, changes in the relative abundance of *Alkalilimnicola ehrlichii* over time for (**C**) HRT of the anoxic reactor and (**D**) sulfide concentration. The changes were determined based on all cDNA samples obtained from six runs of the BD reactor system.

Time-based longitudinal analysis was performed to determine those ASVs that showed a significant change in abundance over time. All cDNA samples from all runs were included in the analyses and revealed that *Thioalkalivibrio halophilus* decreased the most over time. However, since it was only present in low abundance, it was not studied further. The longitudinal analyses of the two most abundant ASVs affiliated with *A. ehrlichii* and *Tv. sulfidiphilus* showed different trends for HRT and sulfide concentration (Fig. 4). At the lowest sulfide concentration (0.2 g/L), *A. ehrlichii* was active throughout the process, but at higher concentrations (0.5, 0.9 g/L), its activity tended to decrease with time (Fig. 4). The relative abundance also reduced with an HRT of 20 min and moderately changed with an HRT of 10 and 30 min (Fig. 4C and D). On the other hand, the activity of *Tv. sulfidiphilus* increased over time at all HRTs and all sulfide concentrations tested (Fig. 4). The steepest increase was predicted with a sulfide concentration of 0.5 g/L. The regression model-based predictions indicated a significant effect of change in abundance with the duration of operation but less or no impact with factors like HRT of the and sulfide concentration of the anoxic reactor (Tables S10 and S11). The residuals for all the analyses can be seen in Fig. S4.

**Fig 5:**
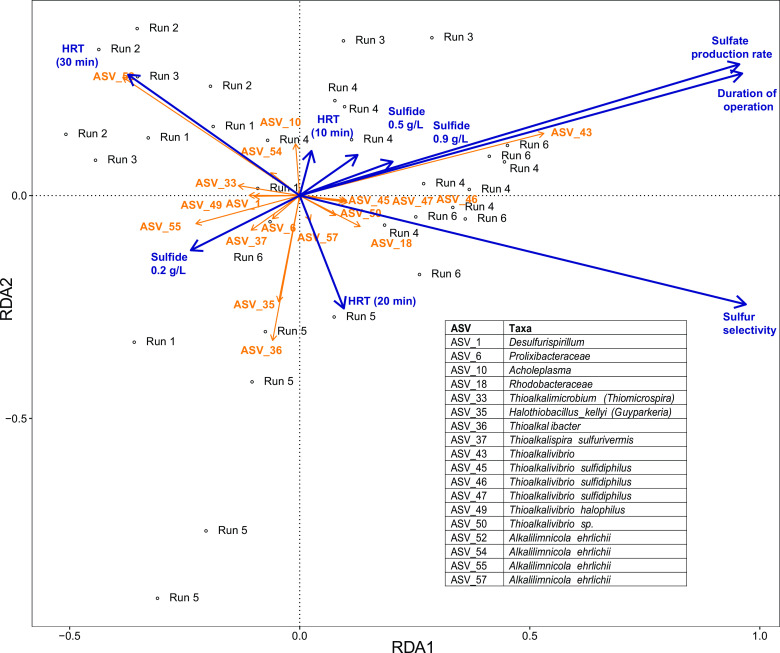
Redundancy analysis (RDA) plot showing the effect of the process parameters HRT and sulfide concentration on the ASVs found in the system. The table shows the annotated taxa for ASVs that have a significant association with the parameters.

To have more insight into the relation of explanatory variables shown in Dataset S1 with the community composition, redundancy analysis (RDA) was performed (Fig. 5). For this analysis, all RNA samples sequenced from the oxic reactor were used. Statistical analysis indicated that the sulfide concentration, HRT of the anoxic reactor, duration of operation, sulfate production rate, and selectivity for sulfur formation were all significant (*P* value < 0.001, *F* = 5.531, and DF residual = 23) and could explain ca. 47% of the variance in the microbial community composition. The air supply rate, biomass concentration, sulfide uptake, alkalinity, selectivity for sulfate formation, and selectivity for thiosulfate formation explain the other 6%. However, these parameters were not used in the RDA due to missing values, multi-collinearity, or insignificant effects on microbial diversity. Moreover, *A. ehrlichii* was associated with an HRT of 30 min, while *Tv. sulfidiphilus* was related to high sulfate formation.

## DISCUSSION

### Experimental runs of the biodesulfurization system

The six experimental runs of the pilot-scale BD system operated at different combinations of HRT and sulfide concentration in the anoxic bioreactor, designed to find the optimal process conditions for good process performance (i.e., high selectivity for sulfur formation). Five out of six runs resulted in >94% selectivity for sulfur formation as compared to the <89% of the traditional process lineup (i.e., without an anoxic bioreactor) (5, 7). The most optimal process condition found was a sulfide concentration of 0.5 gL^−1^ and an HRT of 10 min (Run 3) in the anoxic reactor, resulting in a selectivity for thiosulfate formation of only 0.3% and for sulfate formation of 3%. It can be deduced that high selectivity for sulfur formation is not dependent on the highest sulfide uptake that was observed for Run 4. A continuous increase in the biomass concentration with operation confirmed the presence of actively growing biomass in the system; however, simultaneously, the rate of sulfate formation also increased. This positive relation suggests that with time, sulfide-oxidizing bacteria that can completely oxidize sulfide to sulfate (like *Thioalkalivibrio*) are enriched in the BD system (Fig. S1; Fig. 1A). A recent study has also proposed this relationship and put forward the role of the specific charge of SOB (31). The authors hypothesized that the reduction degree of bacteria decreased, and thus, the oxidation potential of SOB increased with increasing levels of bacteria, measured as mg N L^−1^, and constantly controlled oxidation-reduction potential (ORP) levels (31). In the future, it would be informative to associate microbial composition with the specific charge of SOB. The operational parameters and performance results of the six runs described in this paper were discussed in a previous study (7).

This study aimed to deepen the understanding of the dynamics in the microbiome during these experimental runs and thus focused exclusively on microbial community analysis.

### Microbial diversity analyses

The six runs of the BD system had a total of 544 ASVs, each with 162–225 ASVs (Table S3). These numbers are consistent with the values reported in a previous study (8). However, after removing the low-abundance ASVs, the numbers were reduced to 61 ASVs ranging from 53 to 58 per run (Table S3), suggesting that BD systems do not have a very large and diverse population.

**Fig 6:**
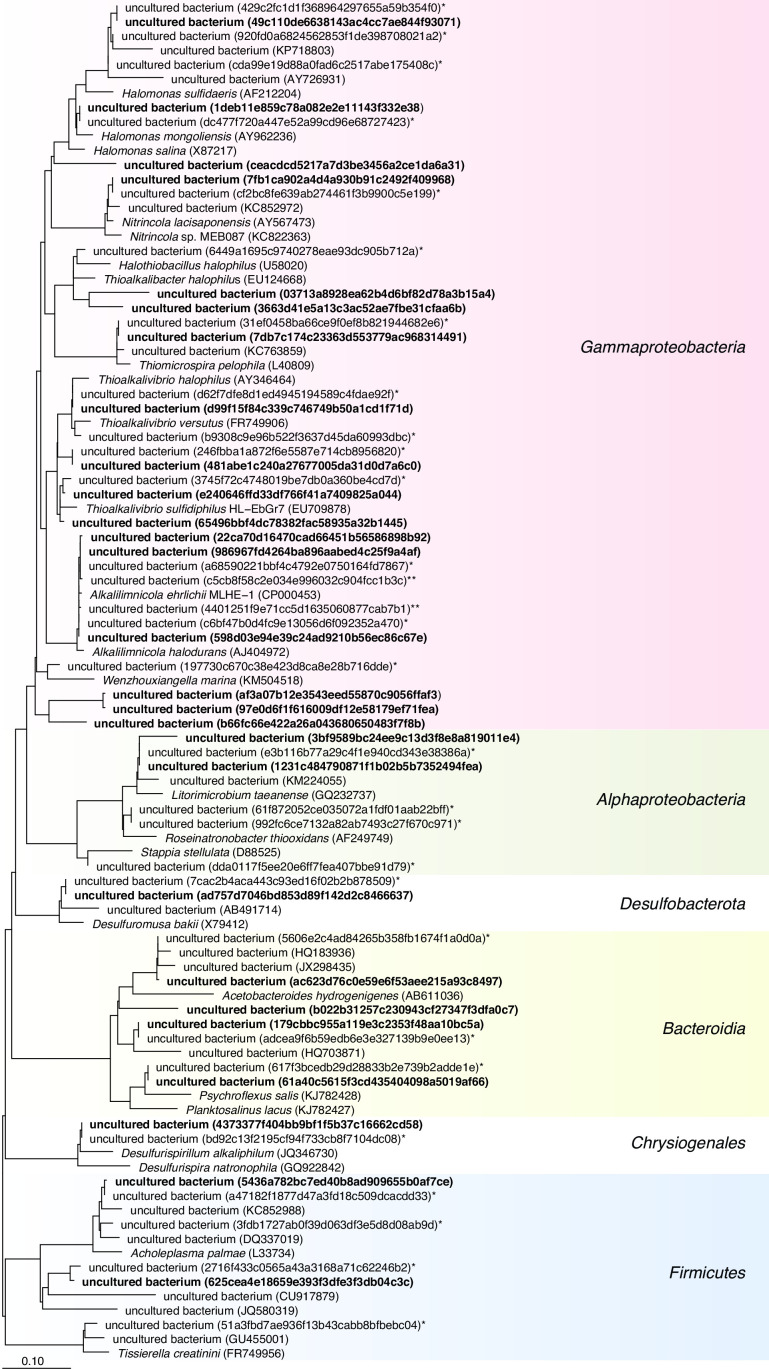
Phylogenetic tree showing the affiliation of bacterial populations represented by the sequences of 27 core active ASVs (Table 4; Table S9) marked in bold. Thirty ASVs forming the core community from our previous study are marked by “*” (8) and other closely related cultures and uncultured bacteria (GenBank accession numbers are mentioned in parentheses).

Alpha diversity measurements indicated that both HRT and sulfide concentration in the anoxic bioreactor played a role in determining the richness of the communities (Table 3). Previous studies have also shown the influence of characteristics related to feed gas on the richness of communities (8). As expected, the number of ASVs in the total and active communities differed, suggesting that not all bacteria that are present are active. The evenness of the diversity, as shown by the Shannon index, was very low and insignificant, which indicates that the BD system does not have a very diverse bacterial community and is enriched with few different types of bacteria (Table 3). This can be because of the extreme process conditions, which enrich only specific types of haloalkaline bacteria. Similarly, richness estimations confirm that the BD system’s three sections have a community of similar taxa (Table 3). It can thus be concluded that BD systems, in general, are less diverse, and their diversity is influenced by the factors that directly affect the enrichment conditions of SOB. This could also be the reason why the members of the core community remain the same across different BD installations (8) (Fig. 6).

Previous studies have shown that the microbial communities in BD systems continuously change with the length of the operation (5). In this study, we also see a drastic change in the microbial composition. The inoculum and the communities at the end of the operations greatly differ from each other (Fig. 1A). The difference was most pronounced for members of *Thiomicrospiraceae,* which were outcompeted by *Thioalkalivibrio* and *Alkalilimnicola* (Fig. 1A). Based on a co-culture study, a commonly found member of *Thiomicrospiraceae*, *Thiomicrospira*, was found to be outcompeted by *Thioalkalivibrio* given its high growth maintenance energy requirement and inability to grow in substrate-limiting conditions (32). In the BD system, low O_2_/H_2_S supply ratio is maintained to promote the sulfur formation. This regime of substrate supply leads to oxygen levels below the detection limit and strong reducing conditions (8). These harsh conditions promote the growth of bacteria with low growth rates and those that can survive the limiting conditions, such as *Thioalkalivibrio* (7–9, 20). The other advantage of *Thioalkalivibrio* species is their ability to store sulfur (9, 32). All the process conditions, together, considerably influence the relative abundances of taxa across the runs in the BD system, as shown by Bray-Curtis-based beta diversity estimations (Fig. 1B; Table 3).

The overall dynamics of the BD system indicate growth and selective enrichment of haloalkaline SOB in the BD system. Furthermore, at the end of the operation, all six runs finished at least one solid retention time (SRT) and were in stable condition. The microbial community in the BD system has already evolved to a microbial composition that represents the functional composition of the system. The results clearly indicate that process conditions could potentially select for certain species, as seen by variation in the abundances of the dominant species *Alkalilimnicola* and *Thioalkalivibrio*.

Across different runs, parameters, both in combination and individually, influenced the relative abundances of taxa across the runs in the BD system, as shown by beta diversity estimations (Fig. 1B; Table 3). At the individual level, the relative abundance mainly differed by the HRT (Bray-Curtis). However, when considering phylogenetic differences (weighted UniFrac), the diversity among samples was influenced more by the sample type. Overall, the results clearly indicate that process conditions could potentially select for certain species and that not all species are metabolically active.

### Active vs total microbial community

The results presented in Fig. 1B and 2B; Table 3 indicate the significant differences between the total and active communities, suggesting that a substantial fraction of microorganisms in the BD system were not metabolically active (during sampling). Looking at the taxa and their relative abundance, it can be deduced that a higher abundance could not be translated into a higher activity (Fig. 1A and 2A).

In the controlled and extreme environment of the haloalkaline BD system, higher RNA-based (rRNA) relative abundance compared to DNA (rDNA) in the early stage of operation can be linked to maintenance and survival for some bacteria. However, eventually, a further increase in relative rRNA would mean higher growth/growth potential. Likewise, the decrease in relative rRNA abundance would indicate a decline in growth/lower growth potential. It can be expected that these non-active bacteria are eventually washed out of the system, and in the long run, only active species will survive and become dominant, as seen for *Thiomicrospira, Halothiobacillus*, and *Halomonas* in Fig. 2A. This was also evident from Fig. 1A and 2A, which point out that only a few genus- or family-like taxa are more active than others and contribute up to 85% of the total population. These taxa are well-known SOB, such as *Alkalilimnicola* and *Thioalkalivibrio*, and others, such as *Lentimicrobium* and *Acholeplasma* (9, 12, 33, 34). At the end of the operations, *Lentimicrobium* was found in runs with low sulfide concentrations, while *Alkalilimnicola* was highly abundant in the run with the lowest HRT (Fig. 1A and 2B). Though very little is known about *Lentimicrobium*, a recent study has reported the enrichment of *Lentimicrobium* with thiosulfate, suggesting its possible role in thiosulfate conversion under nitrate-reducing, anoxic conditions (35). This does not directly imply the BD system but hints at the possible involvement of *Lentimicrobium* in sulfur oxidation.

Given the short HRT of the anoxic reactor (maximum 30 min) and the high doubling time of prominent SOB varying from 2 to 5 h (9, 15), it was hypothesized that the microbial composition does not differ in different compartments of the system. Our study, for the first time, confirmed through analysis that the composition of bacteria in the different sections of a BD system is not significantly different. This was seen for all the runs and throughout the operation of Run 2 (Fig. 2B; Fi[Fig F2]g[Fig F2]. S2 and S3). The observation implies that (i) the pilot BD system is well mixed and recirculated as described in previous studies (5, 7) and that (ii) the enriched biomass in the BD system can withstand sulfide concentrations up to 0.9 g/L and anoxic conditions (reduced to −441 mV). Overall, it can be suggested that in the Thiopaq Ultra system (i.e., including the anoxic bioreactor), more facultative SOB are enriched compared to conventional Thiopaq systems (i.e., without the anoxic bioreactor). A recent study has also shown the sulfide removal activity by SOB in anaerobic conditions, which sheds some light on which SOB could be involved in the anoxic reactor (36).

This study is the first one to report the shared active communities of different runs of the Thiopaq Ultra system and identified 27 ASVs (20 unique genera) as the active core community (Table 4). These active core community members are the same as previously defined core community members in traditional and improved BD systems, except for *Desulfurivibrio* (Fig. 6) (8). The role of *Desulfurivibrio* can be associated with the reduction of sulfur and thiosulfate in the BD system (37). The perpetual presence of these members in different BD systems and under varied process conditions confirms their significance and active role in the BD system, irrespective of their involvement in sulfur conversions (8).

### Succession of the microbial communities

The run with the highest HRT in the anoxic reactor (Run 2) was studied more thoroughly. A series of samples taken during this run (i.e., after 7, 13, 20, and 31 days) showed a distinct succession of the active and total microbial communities (Fig. 2B). The starting microbial community present in the inoculum quickly adapted to the operational/process conditions and changed almost entirely after 7 days of operation. This change manifests the ability of the biomass to adapt to the process conditions swiftly. After 7 days of operation of Run 2, *A. ehrlichii* was dominantly present in the BD system (>50%; Fig. 2A). It remained dominant and active throughout the run, as was also reported in a previous long-term operation of the BD system (5).

Further along the run, a gradual decrease or increase in other microbes can also be seen, which confirms the enrichment of the alternative bacterial community in BD reactors with time. The NMDS plot (Fig. 2B) shows this succession for both the active and total microbial communities. Figure 2A reveals that *Thioalkalivibrio sulfidiphilus, Rhodobacteraceae,* and *Acholeplasma* had a higher activity. The increased abundance of members of *Rhodobacteraceae* and *Acholeplasma* after 1 week can be explained by increased exposure to anoxic conditions favoring their growth (8, 33, 38); however, gradually, they become less active*. Tv. sulfidiphilus* is a slow grower compared to other species and can use sulfide and thiosulfate as a substrate (32).

### Effect of process parameters on the active community

Unlike previous studies that linked abundant taxa and process parameters, here, we investigated these links exclusively with the active communities. The active microbial community from the oxic reactors significantly differed among runs and therefore again confirms the fact that the process parameters have a direct influence on the microbial composition (Fig. 3).

The time-based analyses of the abundance of the two most abundant ASVs, *A. ehrlichii* and *Tv. sulfidiphilus*, showed different trends for HRT and sulfide concentration (Fig. 4). At the lowest sulfide concentration in the anoxic reactor (0.2 g/L), *A. ehrlichii* is dominantly active throughout the process, but at higher concentrations (0.5 and 0.9 g/L), it tends to decrease over time (Fig. 4). The relative abundance also decreased at an HRT of 20 min and moderately changed at 10 and 30 min (Fig. 4C and D). On the other hand, the abundance of active populations of *Tv. sulfidiphilus* increased over time at all HRTs and sulfide concentrations (Fig. 4). The steepest increase was predicted with a sulfide concentration of 0.5 g/L. The abundance dynamics might be explained by other parameters, such as sulfide tolerance, substrate availability, and anoxic conditions. In traditional Thiopaq systems, *A. ehrlichii* was never detected as an abundant community member (8, 39). However, its high abundance and activity in the Thiopaq Ultra systems may be due to its facultative anaerobic nature and sulfide specificity (12, 40). *A. ehrlichii* was isolated from anoxic lake sediment (13), which enables it to survive at the fluctuating anoxic/oxic conditions during circulation. It is known that *A. ehrlichii* possesses both the FSCD (flavocytochrome c sulfide dehydrogenase) and SQR (sulfide quinone reductase) gene complexes for oxidizing sulfide to sulfur but lacks the genes for complete oxidation sulfide oxidation to sulfate (12, 13). The SQR system is more energy efficient (41, 42) and puts *A. ehrlichii* in an advantageous position compared to *Tv. sulfidiphilus*, which is known to have only FSCD genes (10). Furthermore, the *A. ehrlichii*-type strain does not have genes for oxidizing other sulfur species, such as thiosulfate, which makes it advantageous for the system that aims to produce only sulfur (12).

At present, there are no reports that have studied the dynamics of *Tv. sulfidiphilus* and *A. ehrlichii.* However, studies on other SOB showed some interesting findings, such as *Alkalispirillum*, a close relative of *Alkalilimnicola*, which can outcompete *Thioalkalivibrio* because of its high tolerance to sulfide (12). Looking at the metabolic aspect, *Thioalkalivibrio* was initially described as having a low potential to oxidize sulfide/polysulfide and a high potential for the thiosulfate oxidation pathway, with sulfide as the preferred substrate in BD reactors (43). Studies on *Thioalkalivibrio* and *Thioalkalimicrobium* (*Thiomicrospira*) have shown that oxidation of thiosulfate only starts after sulfide is exhausted (44).

Thiosulfate is formed abiotically in the BD system (5). This study detected no or only small amounts of thiosulfate (Table 2), which could be because of a high rate of conversion of thiosulfate to sulfate. RDA indicated an association with *Tv. sulfidiphilus* with the sulfate production rate (Fig. 5) (7). Hence, it can be interpreted that one of the reasons for the increase in *Tv. sulfidiphilus* over time under all conditions is partly because of its capacity to oxidize thiosulfate to sulfate. For the BD process, it would thus mean that *Tv. sulfidiphilus* may not carry out complete oxidation of sulfide to sulfate, as suggested in previous studies (45, 46), and instead use multiple types of substrates to form sulfate. To further lower sulfate production in the Thiopaq Ultra system, the chemical oxidation of sulfide to thiosulfate needs to be decreased. RDA also confirmed the association of *A. ehrlichii* with a higher HRT (Fig. 5). Sulfur selectivity was more associated with *Thioalkalivibrio* genera and, to a minimal extent, with the family *Rhodobacteraceae* (Fig. 5). Unlike in a previous study, alkalinity could not be associated/correlated with the presence of specific bacteria (8), although there seems to be some association with *A. ehrlichii* (data not shown). In a previous study, *Guyparkeria* was correlated with increased sulfate concentrations. However, this study could not see this association with the sulfate production rate (Fig. 5) (8). For *Alkalilimnicola,* more than one ASV was correlated with several parameters, such as HRT and sulfide concentration (Fig. 5). This indicates the presence of different strains of *A. ehrlichii* and again confirms the influence of process conditions on microbial prevalence and metabolic activity, leading to niche differentiation in the system.

Overall, it can be concluded that (i) the Thiopaq Ultra system has a dynamic, active microbial community; (ii) not all species present in the inoculum of the BD system remain metabolically active and those not active will eventually be washed out of the system; (iii) *A. ehrlichii* and *Tv. sulfidiphilus* are the most metabolically active SOB in the Thiopaq Ultra BD systems and these two SOB are the actual workhorses of these system; and (iv) process conditions select for certain species in the system where the selection is driven by a combination of HRT and sulfide concentration in the anoxic reactor.

This study also indicates that total RNA-based microbial diversity analysis is an effective technique to identify the active microbial community from BD reactors. In the future, this method can be used instead of DNA-based analysis. Furthermore, it would be insightful to know which sulfide-oxidizing enzyme systems are more active in certain process conditions. The role of other sulfur species like thiosulfate and polysulfides can also be investigated.

## MATERIALS AND METHODS

### Setup and sampling of the biodesulfurization pilot-scale reactors

Experiments were conducted on a pilot-scale biodesulfurization installation at Wageningen University & Research, Wageningen, the Netherlands. The installation consisted of an absorber column, an anoxic bioreactor, and an oxic bioreactor. Complete details of the process can be found elsewhere (7). A schematic representation of the bioreactor system can be seen in Fig. S5, as also presented in a previous study (7). Six experimental runs were performed to study the microbial communities under different operational conditions, i.e., sulfide concentration and the HRT in the anoxic reactor (Table 1). Details of the operating conditions and how the product selectivity was calculated have been reported and discussed elsewhere (7).

Two milliliters of reactor liquid was centrifuged at 14,000 × *g* for 10 min, and the biomass pellets were snap-frozen using liquid nitrogen and stored at −80°C. The samples for the microbial analyses were collected biweekly from the absorber and the anoxic bioreactor. Samples were collected weekly from the oxic reactor where the actual sulfide oxidation occurs. Samples from the inoculum after 24 h of operation were also collected. All samples were collected in five replicates used for DNA and RNA extractions.

### DNA/RNA extraction and 16S amplicon sequencing

Genomic DNA from the samples was extracted using the DNeasy PowerLyzer PowerSoil Kit (Qiagen, Germany) according to the manufacturer’s instructions. The extracted DNA was purified using AMpureXP magnetic beads (Beckman Coulter, USA) following the manufacturer’s protocol. The purified DNA was visualized by gel electrophoresis to confirm the quality of the DNA. The DNA concentrations were determined with the QuantiFluor dsDNA system (Promega, USA) following the manufacturer’s protocol.

RNA was extracted by the addition of 1 mL of TRIzol (Thermo Fisher GmbH, Germany) and 50 µL of 0.1 M sodium citrate [Sigma-Aldrich (Merck), USA] to decrease the pH; subsequently, bead beating was performed at 7,200 rpm for 60 s in a Precellys Evolution homogenizer (Bertin Instrument, France). Two hundred microliters of chloroform was added to the mixture. After 5 min of incubation at 4°C and 15 min of centrifugation at 12,000 × *g* at 4°C, the aqueous top layer containing the RNA was collected and mixed with 600 µL of 70% (vol/vol) ethanol. The extracted RNA was loaded onto a spin column included in the RNA Purification Kit (Thermo Fisher GmbH, Germany) and purified as described in the manufacturer’s protocol. RNA was quantified using the NanoDrop 2000 (Thermo Scientific, USA). The extracted RNA was treated with Turbo DNase (Thermo Fisher GmbH, Germany) with 44 µL of RNA sample, 5 µL 10× Turbo DNase buffer, and 1 µL of DNase enzyme. The treatment was done at 37°C for 15 min. The DNase-treated RNA was purified using RNAClean XP beads (Beckman Coulter, USA) as described in the manufacturer’s instructions. The purified RNA was quantified with the NanoDrop 2000 (Thermo Scientific, USA), and the quality was checked using gel electrophoresis using an RNA bleach gel containing 1% (vol/vol) bleach and 1.5% (wt/vol) agarose (47). The RNA was then converted into cDNA using 5× *Trans* Amp buffer and reverse transcriptase enzyme from the SensiFAST cDNA synthesis kit (Meridian Biosciences, USA) according to the manufacturer’s protocol. A qPCR using RNA and cDNA was performed with the universal bacterial primers BAC 338F and BAC 518R to assess the level of DNA contamination in the RNA samples (48, 49). For all samples, the RNA samples had exceedingly high Cq values (>22), and the cDNA samples (<10) confirmed no DNA contamination in the samples.

The DNA and cDNA samples (30 µL of 30 ng/µL) were sent for amplification and sequencing to the company MR DNA in the USA (www.mrdnalab.com). At the facility, samples were amplified in triplicate using primers 515f (5′-GTGYCAGCMGCCGCGGTAA-3′) and 926r (5′-CCGYCAATTYMTTTRAGTTT-3′) with a bar-coded forward primer to amplify the V4-V5 region of the 16S rRNA gene (50) using the conditions described in a previous study (8). Purified products were subsequently sequenced on the Illumina MiSeq system.

For the DNA and RNA (cDNA) samples, 2 × 300-bp multiplexed paired-end reads were generated in two batches. DNA and RNA samples from Runs 1, 2, and 3 were sequenced together, and the rest were sent in one set. The paired-end reads were demultiplexed and denoised in QIIME 2 as described in a previous study (8, 51). The four ASV tables and corresponding representative sequences obtained were merged. The ASV table was then filtered with the combined representative sequences to remove the identical ASVs. For specific analyses, the merged tables were filtered as per the need, for instance, (i) table for Run 2, (ii) table for samples that have been sampled for both DNA and RNA, (iii) table for samples for which the samples have sequenced for all sections, and (iv) table for samples from the inoculum and the end of the operation.

### Microbial diversity analyses

Alpha and beta diversity analyses were performed using the merged table rarefied to a sequencing depth of 41,651 reads. Shannon, Chao, and Observed ASV indices were used as alpha diversity metrics, while Bray-Curtis was used for beta diversity analyses. The dissimilarity distance matrix from Bray-Curtis was used for principal coordinate analyses. The diversity analysis was performed in QIIME 2 v.2020.11 (51). Statistical tests, like Permanova, Anosim, and Betadisp, were performed in QIIME 2 to find significant group differences in the microbial communities (52).

For Run 2, section, end of the operation, and DNA/RNA comparison, the specific filtered tables were imported into R (version 4.2.1). Beta diversity analyses and NMDS plots were created using the “phyloseq” and ggplot2 packages (53). The feature counts were analyzed and visualized in R using the package “ampvis2” (https://github.com/KasperSkytte/ampvis2).

### Longitudinal analyses

ASVs that change the most over time were identified using QIIME longitudinal feature volatility (54). The selected features were then analyzed individually using a linear mixed effect model with the duration of operation as a time factor, along with HRT and sulfide concentration as fixed factors as described in previous studies (54–56).

### Core community and phylogenetic analyses

The shared microbial community was estimated using the QIIME 2 feature table core feature plugin. The representative sequences of core ASVs were imported into ARB and aligned using SINA alignment to create a phylogenetic tree (57–59). The sequences of the core community identified in a previous study and other close relatives were included in the tree (8).

### Redundancy analysis

Hellinger transformed feature abundance data from oxic reactors; variables, such as sulfur selectivity, duration of operation, and sulfate production rate, and factors, such as HRT and sulfide concentration, were analyzed in the redundancy analysis. Before the analyses, the normality of all the variables was checked and, if needed, log-transformed to have a normal distribution. The normalized variables were correlated using the corrplot package in R (version 0.92). The analysis was done using the “vegan” package in R. The model’s suitability was checked using ANOVA, and the collinearity of variables was assessed using the variable inflation factor (VIF). The VIF of all the parameters was around two or less. For the selected parameters, an RDA plot was created using the ggplot2 package in R.

## Data Availability

The amplicon sequences are stored in the ENA database under accession number PRJEB58978.

## SUPPLEMENTAL MATERIAL

Supplemental figures and tables aem.01864-23-s0001.pdf Fig. S1 to S5 and Tables S1 to S11.

Dataset S1 aem.01864-23-s0002.xlsx Product selectivity, operational parameters and other performance parameters of six different experimental runs of a pilot-scale BD system.

ASM does not own the copyrights to Supplemental Material that may be linked to, or accessed through, an article. The authors have granted ASM a non-exclusive, world-wide license to publish the Supplemental Material files. Please contact the corresponding author directly for reuse.
