# Supplementary material for: Process conditions affect microbial diversity and activity in a haloalkaline biodesulfurization system
Source: Appl Environ Microbiol. 2023 Dec 11;90(1):e01864-23. doi: 10.1128/aem.01864-23 (PMC10807427; doi:10.1128/aem.01864-23)
Supplement: Supplemental figures and tables — Fig. S1 to S5 and Tables S1 to S11. [file aem.01864-23-s0001.pdf]

Supplementary Material

Process conditions affect the microbial diversity and activity in a haloalkaline biodesulfurization system

Suyash Gupta[1,2], Rieks de Rink[3,4], Johannes B. M. Klok[1], Gerard Muyzer[2], Caroline M. Plugge[1,5]

[1]Wetsus, European Centre of Excellence for Sustainable Water Technology, Leeuwarden, The Netherlands

[2]Microbial Systems Ecology, Department of Freshwater and Marine Ecology, Institute for Biodiversity and Ecosystem Dynamics, University of Amsterdam, Amsterdam, The Netherlands

[3]Environmental Technology, Wageningen University, Wageningen, the Netherlands

[4]Paqell B.V., Utrecht, The Netherlands

[5]Laboratory of Microbiology, Wageningen University & Research, Wageningen, The Netherlands

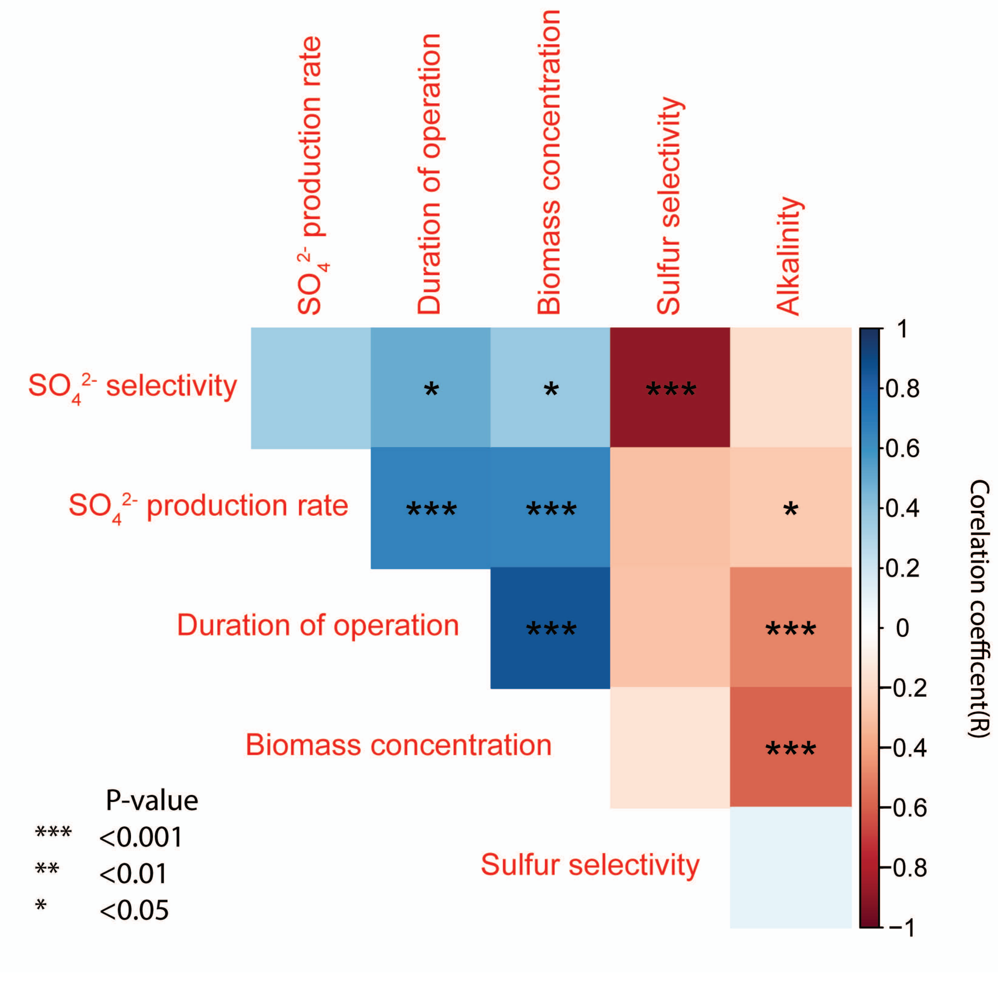

FIG S1 Heatmap showing the correlations between important variable parameters of BD system

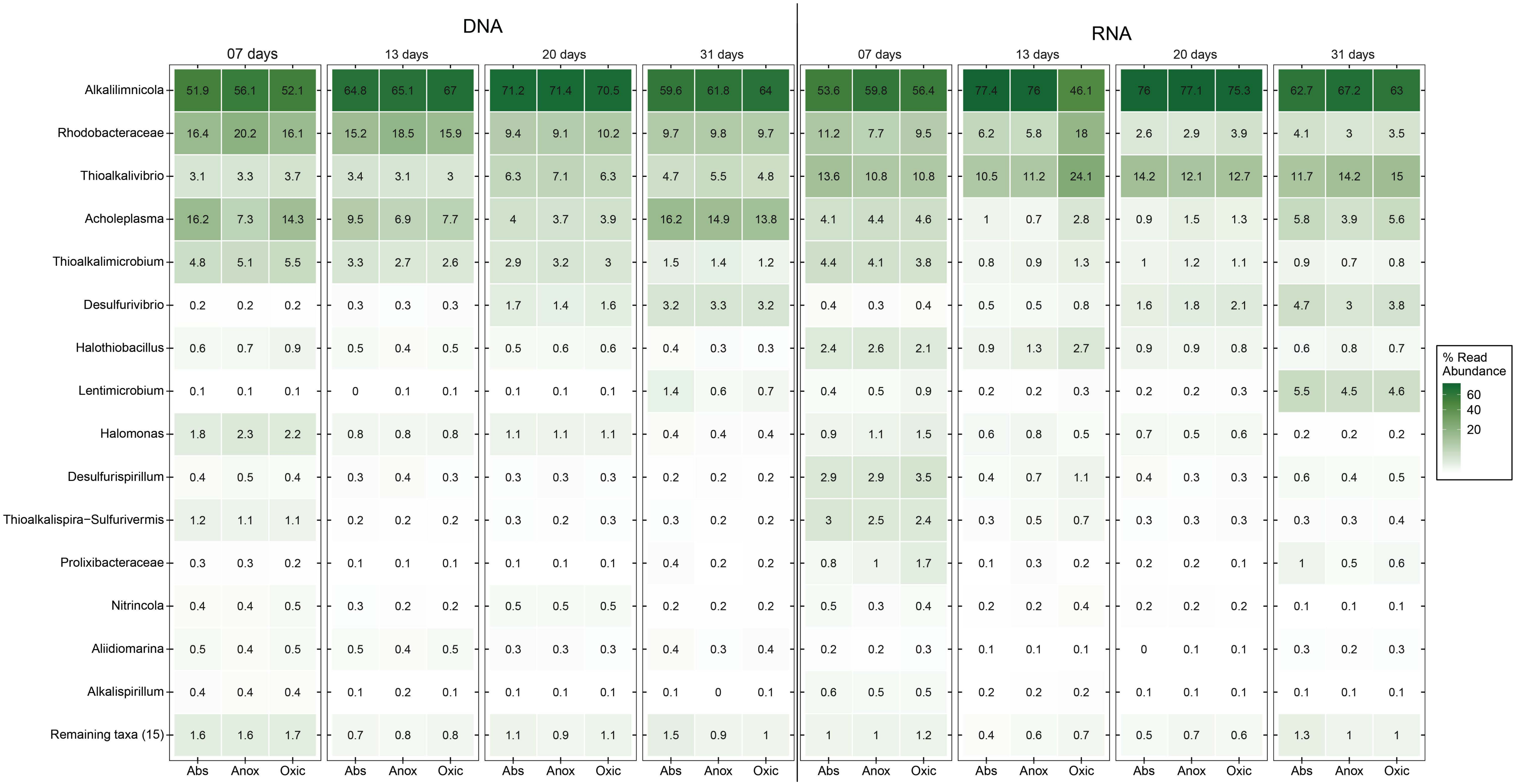

FIG S2 Heatmap showing the relative abundance counts of microbes at the genus-like level for total (DNA) and active (RNA) population at different days for Run 2, for all the sections of BD system. The top 15 abundant genus-like taxa are shown and rest of them are presented as “others”.

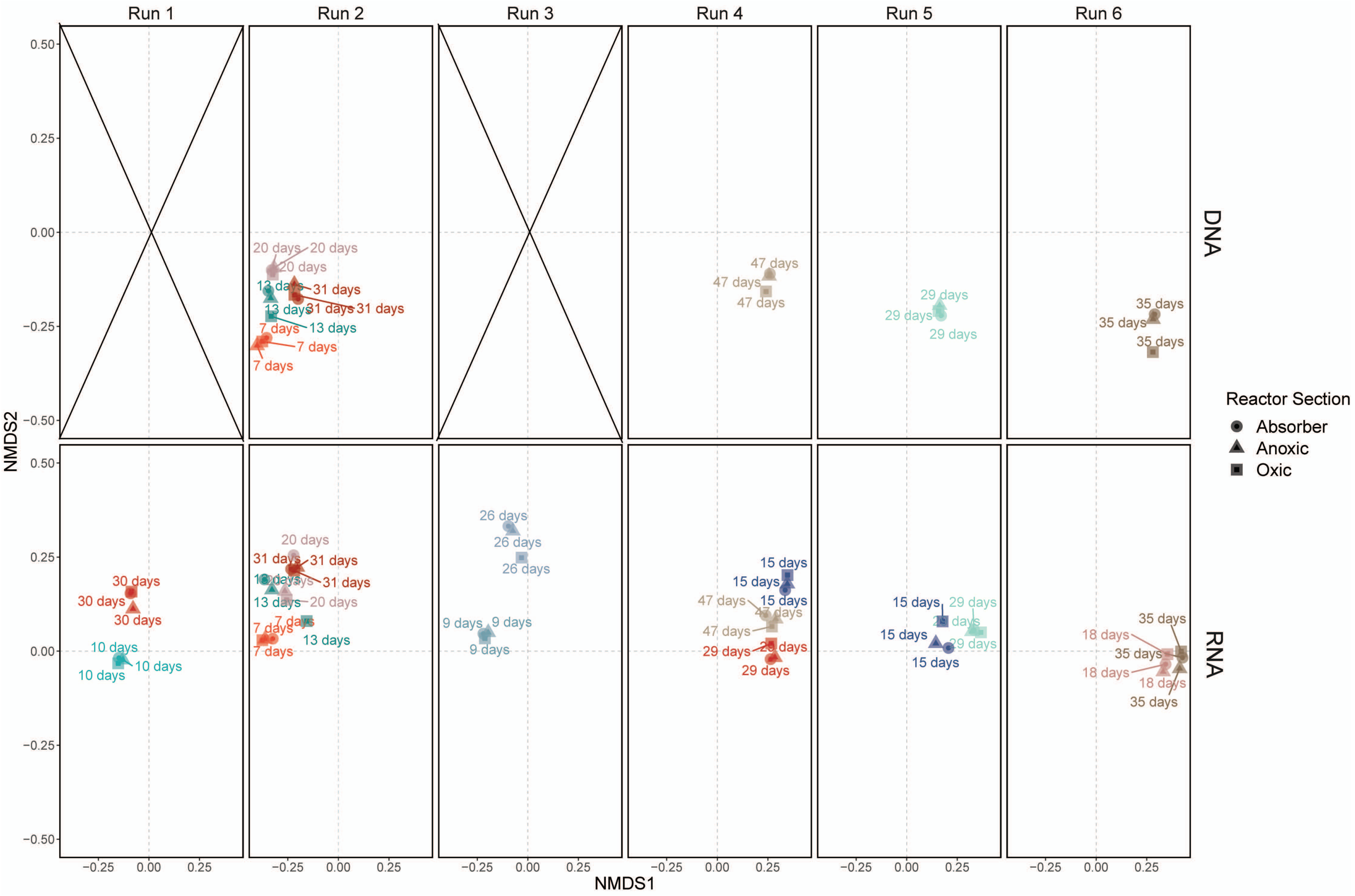

FIG S3 NMDS ordination based on Bray Curtis dissimilarity distances among the DNA and RNA samples from all sections of the reactor at different time points of all the six runs. DNA from Run-1 and Run-3 were not sampled for all three sections.

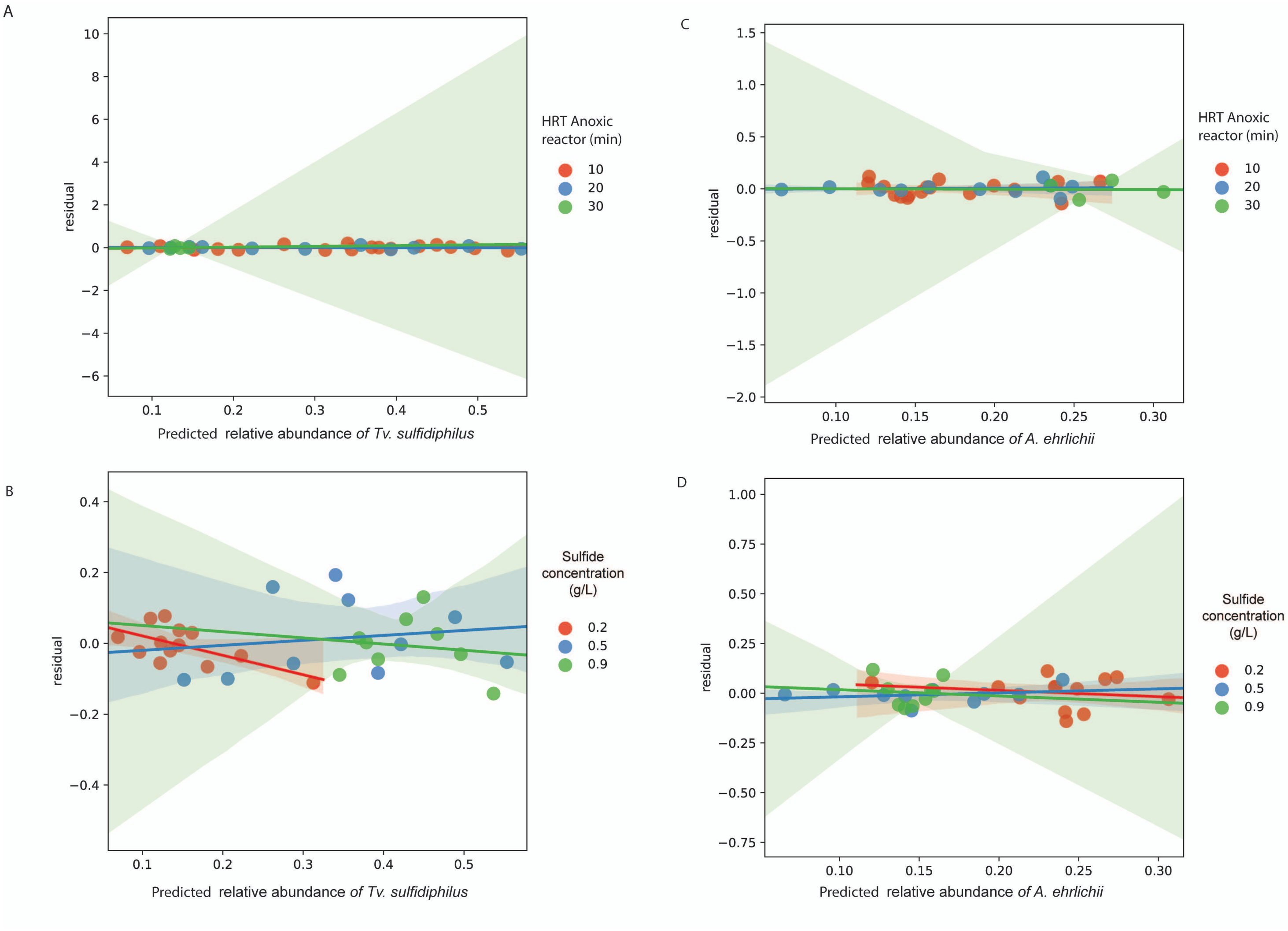

FIG S4 Residual plots for Linear mixed effect models which predicted the abundances of ASVs annotated as *Tv. sulfidiphilus* and *A. ehrlichii* with duration of operation having effect of HRT of anoxic reactor (A,C) and sulfide concentration (B,D)

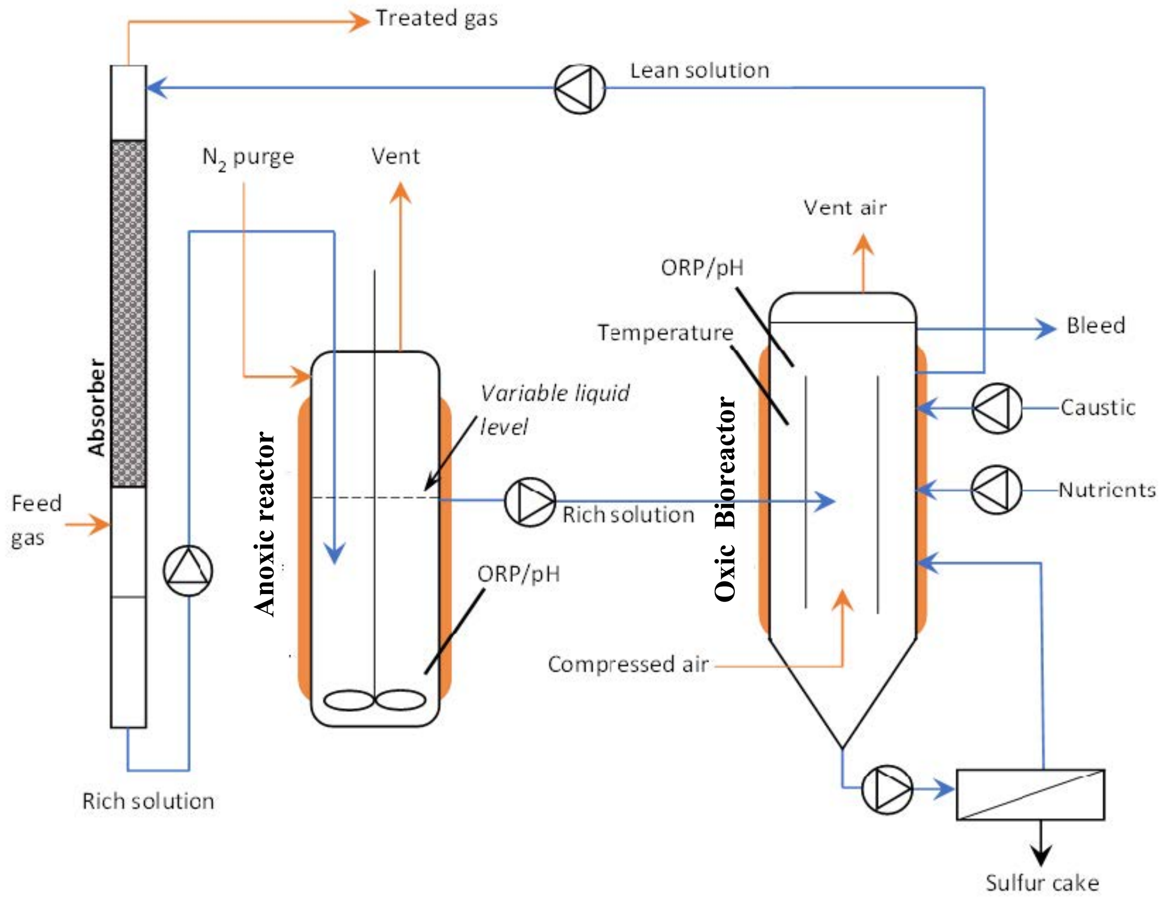

FIG S5 Schematic overview of the experimental set-up, pilot-scale Thiopaq Ultra BD system. Orange lines represent gaseous flows; blue lines represent liquid flows. The figure is borrowed from previous manuscript describing the process operations of the six runs (1).

TABLE S1. Scheme of samples sequenced throughout six runs for DNA and RNA based amplicon sequencing from three sections of BD reactor.

		Sampling scheme					
		DNA			RNA		
	Duration of Operation (Days)	Absorber	Anoxic	Oxic	Absorber	Anoxic	Oxic
Run 1	6			×			×
	10			×	×	×	×
	17			×			×
	30	×	×	×	×	×	×
Run 2	7	×	×	×	×	×	×
	13	×	×	×	×	×	×
	20	×	×	×	×	×	×
	31	×	×	×	×	×	×
Run 3	2			×			×
	9			×	×	×	×
	16			×			×
	26	×	×	×	×	×	×
Run 4	8			×			×
	13			×			×
	15			×	×	×	×
	18			×			×
	25			×			×
	29			×	×	×	×
	33			×			×
	39			×			×
	47	×	×	×	×	×	×
Run 5	6			×			×
	11			×			×
	15			×	×	×	×
	18			×			×
	29	×	×	×	×	×	×
Run 6	1			×			×
	7			×			×
	14			×			×
	18			×	×	×	×
	21			×			×
	28			×			×
	35	×	×	×	×	×	×
Cold store	NA			×			×

TABLE S2 Summary of sequences counts as obtained after demultiplexing of four sequencing projects

	Sequence counts		
ASV table	Total	Maximum	Minimum
DNA_Run1_2_3	2153910	119667	90677
RNA_Run1_2_3	2852684	122712	86886
DNA_Run4_5_6	2817954	119231	83022
RNA_Run4_5_6	4906018	164964	103272

TABLE S3 Summary of sequences counts per sample and number of ASVs as obtained after denoising of four tables and after merging of denoised-chimera removed ASV tables

			Sequence counts after denoising		
ASV table	Number of ASVs	Number of samples	Total frequency	Minimum	Maximum
DNA_Run1_2_3	192	20	1569195	66611	88262
RNA_Run1_2_3	183	28	1920826	57961	78734
DNA_Run4_5_6	283	28	1544039	41651	65229
RNA_Run4_5_6	240	36	2773744	54939	100121
			Sequence counts merge of four ASV tables		
ASVs_merged	522	112	7807804	41651	100121
ASVs_after_filtration	61	112	7754860	41023	99648
			Sequence counts for each Run before filtering		
Run 1	162	12	884609	66611	78218
Run 2	197	24	1780973	62932	86312
Run 3	155	12	824439	57961	88262
Run 4	225	26	1890958	44808	100121
Run 5	179	16	984530	41651	81058
Run 6	184	18	1162306	48105	80951
			Sequence counts for each Run after filtering		
Run 1	53	12	878084	66085	77580
Run 2	55	24	1773908	62503	85874
Run 3	54	12	819247	57805	87990
Run 4	57	26	1881998	44573	99648
Run 5	57	16	973960	41023	80218
Run 6	58	18	1155466	47925	80317

TABLE S4 Table showing the frequencies of ASVs obtained from sequencing project for genomic DNA samples from Run 1, 2 and 3.

ASV	Frequency	# of Samples Observed In
986967fd4264ba896aabed4c25f9a4af	417.847	20
598d03e94e39c24ad9210b56ec86c67e	412.165	20
1231c484790871f1b02b5b7352494fea	221.125	20
5436a782bc7ed40b8ad909655b0af7ce	177.452	20
65496bbf4dc78382fac58935a32b1445	92.521	20
7db7c174c23363d553779ac968314491	69.951	20
b66fc66e422a26a043680650483f7f8b	12.399	20
49c110de6638143ac4cc7ae844f93071	10.891	20
03713a8928ea62b4d6bf82d78a3b15a4	9.991	20
1deb11e859c78a082e2e11143f332e38	9.435	20
d99f15f84c339c746749b50a1cd1f71d	9.163	20
af3a07b12e3543eed55870c9056ffaf3	8.455	20
97e0d6f1f616009df12e58179ef71fea	8.279	20
4373377f404bb9bf1f5b37c16662cd58	7.700	20
179cbbc955a119e3c2353f48aa10bc5a	7.669	20
e240646ffd33df766f41a7409825a044	6.328	20
05bb66f7bb196677ca067d5c19a424a4	5.985	20
58d0e3c2ce468949ea7570310c9cf17c	5.085	20
7fb1ca902a4d4a930b91c2492f409968	4.703	20
625cea4e18659e393f3dfe3f3db04c3c	3.949	20
ceacdcd5217a7d3be3456a2ce1da6a31	3.697	20
b022b31257c230943cf27347f3dfa0c7	3.697	20
22ca70d16470cad66451b56586898b92	3.425	20
83445197c16bee473991239f7e15f11c	3.420	20
9ed27f4bddcb1afb25681e918566a55d	3.409	20
e5046e5c557d1f90925a8ed986a0ef29	3.406	20
12e264fec25bbe1cc51d233b62ccc963	3.353	8
ac623d76c0e59e6f53aee215a93c8497	3.112	20
fcb419099fe77ad65f097cb4bc49a4a7	2.649	20
d51f208ff77a7e9eea303072812164f7	2.499	19
d8f3c828aa33bec6cbd622058dd7e01a	2.270	16
5df82dddb1aa7154e7783d76fbe1ce3d	2.148	20
68146e68feac64537e12a9dd5a2a7e05	2.093	20
e55bb4ef5c583951053e48c2ec554f3f	1.857	20
61a40c5615f3cd435404098a5019af66	1.800	19
dd9e28ed02dfba5d507fadcf492d6713	1.706	14
831a520fbfce915b3d2081449e9aa0a7	1.695	19
77ac02c2d6e75ac848fd96f113337ca0	1.671	19
5a71155f614b32e16173c607d01764a9	1.403	14
33cd3dcab2602b1bb11a77c58850e841	1.294	20
4541d7f9536c04dd1151a0be0ceee228	1.160	11
835c3e0ccf6a06b3ace58c9c6bae1fbd	919	15
9e8a870c7a9c57f3d3b734318536d7f0	896	8
a2d76b570fa0469135640884599a2af2	833	20
6d23cba5806cb0cd964a510619774732	792	7

6c55ab68cd43b9974908c956a8a6ac9d	787	20
01591a6731e83bd7b04c607d8b6632eb	718	11
7624e20a982ba894a211695066600830	697	19
ad757d7046bd853d89f142d2c8466637	645	20
bce3d14531e0e7da2878fcf566a19046	631	20
3663d41e5a13c3ac52ae7fbe31cfaa6b	571	20
9b028b89b8392d8f3d09fe92fb8f2b98	550	9
a39f3d8925ca6d49ab17fc8b1a333c1a	536	18
8bcc33c1e592d4673edcf002cb0eb3c9	507	17
34363eab7d41653ba9f4b878f109ab34	495	7
481abe1c240a27677005da31d0d7a6c0	477	16
80e52039c3b6d220ee9b548770984f41	460	7
2808f5f8c95fb0018212221170abbddc	419	19
cc231c20ce7de17921d5e22f37ee0fcc	385	19
0218d72d766f03106b4db2198366e75a	257	14
20690749005b354b43360bc864f35ab3	243	13
fd5c4af273a2cddec97f31800dd1ea2b	242	16
a1bc050e9ebf69966eaf6e4215f53353	225	10
c3d4dc2a71f4e938cf14444eab2fb323	222	18
6297cf382b94ea9f464a72a995fea3c0	205	9
238384ca735cb21dc3a3209a984f7de5	194	12
3d2404d3c57e42005c0a4658ae53f621	179	20
5370ffbc2f778289e48d62501b271305	166	2
3982b3368d72d1c51207dfbb6cbe24d8	161	3
a9030113cda02ce233b9b23511dff2a7	148	9
85cd518ae4e18c79a387593c8290fa76	110	13
0d5a9abf700cba2f3041046e57158f5d	108	7
6c5a6031a86bcd5b3e9e0e7333112c39	108	3
6c8d86a415057405c674b00bf950dee2	103	9
ee685de82a7ed29df93f98fcb171bd84	102	8
1ddf6bed8b8d2bf886a07e2078241e9e	89	10
cc3d5fb92638a2314ef5c5e3dd9d34b7	89	3
908b204b1242810169c973717e22e148	85	6
6e0e305a267f3644589dd48086a5c7cf	81	9
19c250b38d235c3d05c4d77a534db8f8	74	2
1c02fbe4ea1fab6a1f4f65eeb3515d52	64	5
9e6201146e8f882cb2efcd6ec66bdf5e	61	6
c9b2ec35adcd43be7214c370052968fa	56	7
2ffea2b1f0e29444ce8955bbe8fedc7f	56	3
7a201de781d4615f992a2fd876dbd375	55	4
1a87be1b3fd874307d6334aa57e432f6	55	5
23e6778d3d7687ab4c7d876f6cbd8973	51	3
9ffb8551e2a8eda9ec80d31fb0ede081	44	1
8585dece87d47c8062cabe98ffe7980d	43	1
5e0fb96dc99ad1e004c6467e9ccd2a52	40	6
2a0cf99f0b2cfdf330e5f9408bc2d734	40	2
2f09415dff8d1737f55e774fa5a84b8b	38	7
aae9b9a6fb6d77234c967bf45dce5196	38	3
91d20ce368b224958b151ff03440ef98	38	5

14a9ba70528cd8ad6c741bcf33bcb377	38	4
7cea1277e914a269e74cf3e0d4297d05	35	4
376e72395ed71165c82e66b4716d831e	35	4
19bb4b1d639a45b8d6483ef7625508cd	35	3
6ed527434810e165be16c9c95c47c5d2	34	1
cc71ec7192f81b0421f40c33862346b5	34	1
efc1610ee23b8fd2b868d52265f7ba75	32	2
c121eb4aeed68693639c6c97aa731ce7	29	5
45b29c57b9f166e67685c5379120dbcf	27	2
2332e8722d90259636448e1e82d9113e	27	2
66713f4eabc4899e97620b42c41863da	26	3
0c19336826fc10c8eac37262dc539622	26	2
a90bf1263930109ee68c95c31c42ad24	26	4
05d1122ee53417052ed32e92e58d309e	25	2
57c34d52c453f938ab74b0cb3aab9583	25	2
ab39860d7aad8f55ff2e37cf4679cc1e	25	1
02bfbf94e7a6e52f2ff78a7b09a86d83	20	1
2d0318b452ca34384b89875743824048	20	1
4ac7d3006cd4da603a5d3cab61249f7f	19	1
eb9a53bb5e4baa670f0c3b3f960deebb	18	3
fb91cdba406d4a0b4ff9da4c2872ff31	18	1
6c18638076676fa55fd3a4f8c3449f56	18	1
3f9e45056e4bff201076d5da60d19141	16	1
ae44199500a38ef76fe56332d6ba2d7f	16	3
f599a769f4c898cf494f7e76c35ed91c	16	1
63027a8f109a3a591fe6c6dae22b10cb	15	3
98da7094bed979c123fd34abc082b3a7	15	1
2e13a15354eb43c0775fcd790894a461	14	3
e4fdb08c6bdb194ee9bd4a80de51ce0f	14	1
b4856372de34260b5e8f83360eb40b4f	13	1
4d07c4262b7e7e40dedd88d23e7f224e	13	2
99e03611eb198d71cd951009235ef31a	13	1
4d2d0f7bee11667ac420f44e1cb8b5f5	13	1
1666bcd414a0fcd8881a4fb5ba9ff702	12	1
905ef8101f26383dfeb299eef0f7b6f9	12	1
968e041482eecf7c6ed0ad3e787b5d9b	11	2
57bb4e11927bb0dd9b48ec8fa0230205	11	1
51dead87dccaa7c310653c364dd76fae	11	2
d14ef7597f581525ec2607e6b591f12a	11	1
a07bc1b502bc0666b7e0f9ae35626391	11	1
a871e00cb5c460c97704ea57519c52e9	11	1
4fc4b46df175d368659d622d8288ed16	11	1
4479e6593cd34f2983abaf28bd59aee3	11	2
4f2d6ebf40c3a9d7c5c969b6d5491f3d	11	2
9056a14ab8d483816142f9552d088377	10	1
3e21fc01f9867bb23868480f19006788	10	2
da003c5b80bcce6c7867a28ac3b2e9e6	9	2
1e22e775a1ac07f39ba599d6683b4e2f	9	1
a22ce57580bc2220580ebf9909410777	9	1

e1d6be8ad9b33b0cf8c6670a58d4fe02	9	1
e32f54287d83be049632e61f9e6fb091	8	1
eab937ecd354d0c94fc1f94db4c0e1ba	8	1
d70c5d0b986bf2447fed1cb9a542da18	8	1
1b4b73ca73a28ac494503a1b1daad14a	8	1
d5e68d412750a55cbb60e0dc87090e27	8	2
e7ff57b2d8a75972c5a74082f9fea66d	8	1
4dd9f814152f6b2d917ee64f6d7880d8	7	1
2c53067d70304de50cfbb8136fe1635e	7	1
f7f2737b8e1459c317ffa3c4ebe126bc	7	1
7fb24aa3bf5c45149195831a5b84ed17	7	1
87a9d09285ca978c6c6bade91d308af9	7	1
30b31fb66650424d81a9bd6f60224000	6	1
d7dbe417ddb2087201bd9549ea0eccdc	6	1
b52531c4c3f348cbf87603e83dc75664	6	1
1ac49d7edd5494969e82e42526aa1d0e	6	1
fd35ee091a1df44c4cc0ac566689e383	6	1
f7c006c1694350e670d91f68e1697f8a	6	1
235489e885312c5a8524ea48fa4252e2	6	1
cc5298164c630fb73fff1dd1714caa31	6	1
6593ba4d1e51c31ef13d87c5016f4a32	6	1
4c0191d947cbf14d60855ae5de0d3ac8	5	1
850dea80c7bb496ac3388d2756b784fa	5	1
08e4f7408eb3821919acab4dca725c65	5	1
7201d37dcbd03148a6e97c8fbb81423f	5	1
a4a5656e4cf33523cec6b4b495d5082b	5	1
de39782dafa438932421b40ead1a639b	4	1
675aa026dcd120f995ea7e16285f6e90	4	1
e6027d060759c2593d9813cf8a7121cd	4	1
e410046b700a620166ee3a172edc9564	4	1
5d4bd876d644427e8865fcfe49c7fabc	4	1
eb9a6ff1641504d8b72d679bca7ee599	3	1
8c86684d1e18071691e6c92418ddbf02	3	1
6944be8af712798697e0a4b34b17d1c6	3	1
9925bea90bbc9b7a2c5f0bc33018e58e	3	1
fed82b18e87015d480f9f30d646f9761	3	1
c93339eedc52a59a6e584cb3725027b6	3	1
6a927ff6420d4618b6056b190d28328c	3	1
3a2c6e082cca7094d01a91ff5f1c4463	3	1
6c2bb40982eab12e7efde4132ca2d1a5	3	1
99c0ae36ffbf583cf77c1f4c9848c949	2	1
36ac123fed14bdb910433dcd22b9c921	2	1
ef66ec977ae8df1fd6bd734fec4c8117	2	1
c6655e607954b0c5b082f47527a10529	1	1
8f918ef9b5c7347caf6f717c7e9015a9	1	1
fd990c15ddaff376ceda14eb94069e3f	1	1
3184b5d04dfdb737d5ecaea90f070c9d	1	1
67aa33d2f3f095e8e1075e6228dd96e6	1	1
cc0ab98c0077d46b69172f7d0c335bcd	1	1

TABLE S5 Table showing the frequencies of ASVs obtained from sequencing project for RNA (cDNA) samples from Run 1, 2 and 3.

ASV	Frequency	# of Samples Observed In
598d03e94e39c24ad9210b56ec86c67e	472.468	28
986967fd4264ba896aabed4c25f9a4af	438.230	28
65496bbf4dc78382fac58935a32b1445	347.392	28
1231c484790871f1b02b5b7352494fea	171.113	28
5436a782bc7ed40b8ad909655b0af7ce	99.244	28
179cbbc955a119e3c2353f48aa10bc5a	62.642	28
7db7c174c23363d553779ac968314491	56.206	28
b66fc66e422a26a043680650483f7f8b	35.566	28
4373377f404bb9bf1f5b37c16662cd58	29.029	28
03713a8928ea62b4d6bf82d78a3b15a4	28.343	28
e240646ffd33df766f41a7409825a044	21.588	28
d99f15f84c339c746749b50a1cd1f71d	20.452	28
b022b31257c230943cf27347f3dfa0c7	15.779	28
61a40c5615f3cd435404098a5019af66	12.472	28
97e0d6f1f616009df12e58179ef71fea	12.466	28
af3a07b12e3543eed55870c9056ffaf3	12.446	28
49c110de6638143ac4cc7ae844f93071	8.042	28
1deb11e859c78a082e2e11143f332e38	5.723	28
83445197c16bee473991239f7e15f11c	4.676	28
22ca70d16470cad66451b56586898b92	4.219	28
7fb1ca902a4d4a930b91c2492f409968	4.146	28
9e8a870c7a9c57f3d3b734318536d7f0	4.107	22
ceacdcd5217a7d3be3456a2ce1da6a31	3.020	27
4541d7f9536c04dd1151a0be0ceee228	2.948	17
625cea4e18659e393f3dfe3f3db04c3c	2.922	28
5df82dddb1aa7154e7783d76fbe1ce3d	2.897	28
3663d41e5a13c3ac52ae7fbe31cfaa6b	2.883	28
3bf9589bc24ee9c13d3f8e8a819011e4	2.482	28
12e264fec25bbe1cc51d233b62ccc963	2.434	9
05bb66f7bb196677ca067d5c19a424a4	2.427	28
d8f3c828aa33bec6cbd622058dd7e01a	2.285	19
fcb419099fe77ad65f097cb4bc49a4a7	2.064	21
a2d76b570fa0469135640884599a2af2	2.035	28
68146e68feac64537e12a9dd5a2a7e05	1.876	22
d51f208ff77a7e9eea303072812164f7	1.803	16
481abe1c240a27677005da31d0d7a6c0	1.622	27
ad757d7046bd853d89f142d2c8466637	1.471	28
e55bb4ef5c583951053e48c2ec554f3f	1.470	26
33cd3dcab2602b1bb11a77c58850e841	1.330	28
dd9e28ed02dfba5d507fadcf492d6713	1.149	15
ac623d76c0e59e6f53aee215a93c8497	1.118	27
cc231c20ce7de17921d5e22f37ee0fcc	1.107	28
5a71155f614b32e16173c607d01764a9	1.075	12
1dadaaefef4d77778b805052d119f82a	742	11
835c3e0ccf6a06b3ace58c9c6bae1fbd	735	18

c707860eb7d75197d4bffc989187fe71	722	25
cc3d5fb92638a2314ef5c5e3dd9d34b7	701	11
fcacbc2a90b3e158762926d9ab3178ec	654	12
4fc6daa94b75fb73fe80656de5730631	596	11
58d0e3c2ce468949ea7570310c9cf17c	555	28
8bcc33c1e592d4673edcf002cb0eb3c9	537	18
efc4bb5dc12fcef552cfaa5d4ea1cb61	527	22
238384ca735cb21dc3a3209a984f7de5	429	14
7624e20a982ba894a211695066600830	419	17
6c55ab68cd43b9974908c956a8a6ac9d	414	25
20690749005b354b43360bc864f35ab3	355	21
1a87be1b3fd874307d6334aa57e432f6	314	10
3e21fc01f9867bb23868480f19006788	299	9
6d23cba5806cb0cd964a510619774732	245	2
19c250b38d235c3d05c4d77a534db8f8	236	4
91d20ce368b224958b151ff03440ef98	232	17
6c5a6031a86bcd5b3e9e0e7333112c39	193	3
e121db2eb399a18856cfa05ca284c08f	191	5
21ebdde4af3aea36188cb9eeb81eb154	183	4
908b204b1242810169c973717e22e148	179	13
2332e8722d90259636448e1e82d9113e	129	6
831a520fbfce915b3d2081449e9aa0a7	120	10
a4761f7c93d515df40deab14b48777bf	119	5
a1bc050e9ebf69966eaf6e4215f53353	117	9
a9030113cda02ce233b9b23511dff2a7	113	7
a07bc1b502bc0666b7e0f9ae35626391	102	2
19bb4b1d639a45b8d6483ef7625508cd	88	3
57c34d52c453f938ab74b0cb3aab9583	78	3
6e0e305a267f3644589dd48086a5c7cf	76	8
01591a6731e83bd7b04c607d8b6632eb	71	6
5bea5cfdb8bff735befd718017ea1a8c	70	4
f57e6a8686e524991a4da35eaedb5c7a	63	1
c3d4dc2a71f4e938cf14444eab2fb323	62	7
ceed4dc4dddcce320ea6b073db284644	62	4
86692a37af0ba511cf79c70f28732e95	57	7
0335a90f3710821afb3afaf9e3167868	54	7
181e18e15d37c16cafc4a46eceef6919	50	3
812e739c966ac77def6a2623efc0b848	49	1
a39f3d8925ca6d49ab17fc8b1a333c1a	48	5
20cf65e815e5dbb356c800fc92f03676	44	1
bce3d14531e0e7da2878fcf566a19046	42	6
31dcb2b2171013304ef5ea5ad17e3e55	41	3
cc5298164c630fb73fff1dd1714caa31	40	3
e6027d060759c2593d9813cf8a7121cd	35	6
4d2d0f7bee11667ac420f44e1cb8b5f5	33	2
cf799fb75c2c256239b2567d14f5e357	32	5
00a330dbadc40c961ed97fb3d88897bc	30	1
ea1769adc4181375706eecd97394b8c0	29	5
fd5c4af273a2cddec97f31800dd1ea2b	28	4
80e52039c3b6d220ee9b548770984f41	23	2

faf23e14bc07d8c09491f3361f71d807	23	1
946c3853f952f96ab2811a0c046462d5	21	1
3bfefdea4f56503bb045813dc77a5179	21	1
12b85924c194c76cfa9c7d89ce9376e5	18	1
5e0fb96dc99ad1e004c6467e9ccd2a52	18	5
03e699a77cf344b1ba5a0d40b4ce97ef	18	1
7515e6d25268ec6b1cf71862c597e663	17	1
b4856372de34260b5e8f83360eb40b4f	16	1
2808f5f8c95fb0018212221170abbddc	16	2
9bcf746120a35ec999eebf145e168e30	16	1
a113d5803b9ab120ed8651984f0fee72	16	1
7f55b63cb38020db11785af2393472d9	16	2
2161fd52308152215dcdec19702907d5	15	1
994db2eb36da4b53de581daf4ad9e2dc	15	1
b439f154aca81bfde353580eb801ee4e	14	1
1f48d06fde0de8a1805385cf2c1ec529	14	1
86b091d4fc7f9a87735339bb72502026	14	2
5d4bd876d644427e8865fcfe49c7fabc	14	1
a421ba0dcd3ce10ece9a8f495adb3faf	14	1
77ac02c2d6e75ac848fd96f113337ca0	14	1
c4677339dd0a745c23597cc60f29ea24	13	1
42bee138c83cdda22dae6c8e31678f10	13	1
331e47bc95931574576e67e0b4738697	11	1
04f42921bdf8c6ba78d3dc9d08dbaff6	11	1
7201d37dcbd03148a6e97c8fbb81423f	11	2
2c3d88d72198fa47b8f8c9270cc56485	11	1
df635d0ebc5626f0d25c492d875c65ef	11	1
0fe676474cb62e5781a215eeb66b895a	10	1
12dc019695ba2d4d8f648eef6b0d3896	9	1
b1299a1ca4669ebf91e7d4053be86b35	9	1
e3195d003899773618689ae607fd7b99	9	1
42f7e686e76739283cbb4fef7a570aa0	9	1
da003c5b80bcce6c7867a28ac3b2e9e6	9	2
c59a118afdc652913766de4b1753f102	9	1
4bdffa859d3f9098f6fa14ee1afc96e5	8	1
19d1e2652e6ebff7e8ca2b4ab2942548	8	1
cc71ec7192f81b0421f40c33862346b5	8	1
05bfcca37a7eed3ede4a1d804d33f8f6	7	1
c4c424c3c7da1c004ded51bf948f8900	7	1
260e85089fc5e09c2e31f03d458b4cb0	7	1
9f1e537ae1b81eba9129f16da13f8fe0	7	1
2f09415dff8d1737f55e774fa5a84b8b	7	1
38a43e4aab5a8b8b44fa19f441707e15	7	1
0a2ada1ea7f4be8361ccea4dbbf40777	7	2
3184b5d04dfdb737d5ecaea90f070c9d	6	1
02b3f5a0f26f23a214129736106baac1	6	1
9e137da4097f161ff4648760463590dc	6	1
701b5245cc04ce7077d6b9ab549364b4	6	1
6c8d86a415057405c674b00bf950dee2	6	2
4954c6218cb7f3d9160d63d24a464dc1	6	1

9e6201146e8f882cb2efcd6ec66bdf5e	5	1
089e857d1123f321ab3912868a9c3de9	5	1
66713f4eabc4899e97620b42c41863da	5	1
da60bcf77d2a10e08f2404f3803a12bf	5	1
05605f5508068d8a724d42360ec1a8f1	5	1
fe68b7957842ec0d4d3ca4bdaf4fe471	5	1
14caed348f1f188683986fc601c34c7c	5	1
eb9a53bb5e4baa670f0c3b3f960deebb	4	1
73ecbe31062a71a3019b565882bc8b6c	4	1
b3b06dbf37a7eab99bf7ae548c4c0d2b	4	1
7b8a4fc45dc71da91d46d77f2524226e	4	1
d8a018f796c5c7043395c6ac1844ab21	4	1
02ff464f2aeb1496df003693b2516c7c	4	2
9131e973087f8e35822b3f2f24d040e4	4	1
c1fa86c28a672d3d1e154fa45acfdb9b	4	1
c49a073bdcd7232774f9eb14b7db92e1	3	1
2c53067d70304de50cfbb8136fe1635e	3	1
5ffab526b6d78b4142b8d0da3f3ee025	3	2
c21b68031aec4cb9b53987d9bf93babf	3	1
8b84d4f82dbc4cb715e7a7a6e9e5b846	3	1
99e03611eb198d71cd951009235ef31a	2	1
7967684498524f06c6bcdff2c327aa5a	2	1
05f3e6817f0b313564e50a1502e29ff6	2	1
58be2b3bf6af851bf0969d74b8bec83a	2	1
2307bde932214068ba9462f35763404a	2	1
c733f0e80f8196c1b45924f765cef08b	2	1
0e41e5c44d294b1cc181b68a7289ab08	2	1
76bd6b327d8ee5bdc8475077809e7bb7	2	1
376e72395ed71165c82e66b4716d831e	2	1
14a9ba70528cd8ad6c741bcf33bcb377	2	1
75cc0cfb66231e69a8b8867db444a7e9	2	1
c417cde5e4588de1e788a0095a238a89	2	1
fce3b10b4652e4d04f9bb634c4419ca5	1	1
dde7cd6b574df17c17f0f1b6362d3037	1	1
0ea8fddd7b324cf89c2f8b451e341778	1	1
947c443d5fd8a2d721cb383bb070a04a	1	1
78679e4bd376d7e98ea29b6ea7e6b15b	1	1
7415ff16f1d0d244db13ba32b87ebba0	1	1

TABLE S6 Table showing the frequencies of ASVs obtained from sequencing project for genomic DNA samples from Run 4, 5 and 6.

ASV	Frequency	# of Samples Observed In
1231c484790871f1b02b5b7352494fea	312.899	28
986967fd4264ba896aabed4c25f9a4af	311.308	28
65496bbf4dc78382fac58935a32b1445	255.570	28
5436a782bc7ed40b8ad909655b0af7ce	173.674	28
598d03e94e39c24ad9210b56ec86c67e	102.382	28
12e264fec25bbe1cc51d233b62ccc963	51.553	28
7db7c174c23363d553779ac968314491	48.396	28
03713a8928ea62b4d6bf82d78a3b15a4	28.588	28
e240646ffd33df766f41a7409825a044	22.506	28
179cbbc955a119e3c2353f48aa10bc5a	18.512	28
af3a07b12e3543eed55870c9056ffaf3	13.515	28
97e0d6f1f616009df12e58179ef71fea	13.172	28
b66fc66e422a26a043680650483f7f8b	9.240	28
3663d41e5a13c3ac52ae7fbe31cfaa6b	9.001	28
49c110de6638143ac4cc7ae844f93071	8.756	28
d8f3c828aa33bec6cbd622058dd7e01a	8.614	28
ceacdcd5217a7d3be3456a2ce1da6a31	8.595	28
5829f592447e5b0d8784f43150ffb56f	8.168	27
d51f208ff77a7e9eea303072812164f7	7.752	26
d99f15f84c339c746749b50a1cd1f71d	7.614	28
e5046e5c557d1f90925a8ed986a0ef29	6.887	28
1deb11e859c78a082e2e11143f332e38	6.880	28
22ca70d16470cad66451b56586898b92	6.487	28
61a40c5615f3cd435404098a5019af66	6.310	28
4541d7f9536c04dd1151a0be0ceee228	6.244	28
9e8a870c7a9c57f3d3b734318536d7f0	5.792	28
b022b31257c230943cf27347f3dfa0c7	5.784	28
58d0e3c2ce468949ea7570310c9cf17c	5.457	28
7fb1ca902a4d4a930b91c2492f409968	4.454	28
dd9e28ed02dfba5d507fadcf492d6713	4.368	25
4373377f404bb9bf1f5b37c16662cd58	4.165	28
5a71155f614b32e16173c607d01764a9	4.056	24
ac623d76c0e59e6f53aee215a93c8497	3.997	28
4b347b76b57c2e71705057dd9dd1aed9	3.765	23
e55bb4ef5c583951053e48c2ec554f3f	2.864	28
831a520fbfce915b3d2081449e9aa0a7	2.645	25
2332e8722d90259636448e1e82d9113e	2.640	27
625cea4e18659e393f3dfe3f3db04c3c	2.155	28
769cf91f760ccf78dfd1a4e0fbbb0b76	2.045	13
1c9d49371cb8032609739d5de05f50e5	1.932	11
5df82dddb1aa7154e7783d76fbe1ce3d	1.800	27
01591a6731e83bd7b04c607d8b6632eb	1.571	12
33cd3dcab2602b1bb11a77c58850e841	1.411	26
05bb66f7bb196677ca067d5c19a424a4	1.340	27
80e52039c3b6d220ee9b548770984f41	1.321	15

9b028b89b8392d8f3d09fe92fb8f2b98	1.320	6
6297cf382b94ea9f464a72a995fea3c0	1.280	16
6c55ab68cd43b9974908c956a8a6ac9d	1.249	24
a2d76b570fa0469135640884599a2af2	1.229	26
16180afbe4300d9862ebcc81f3914397	1.178	7
68146e68feac64537e12a9dd5a2a7e05	1.030	19
7624e20a982ba894a211695066600830	1.007	19
77ac02c2d6e75ac848fd96f113337ca0	821	16
ad757d7046bd853d89f142d2c8466637	785	28
34363eab7d41653ba9f4b878f109ab34	676	8
fd5c4af273a2cddec97f31800dd1ea2b	669	22
bce3d14531e0e7da2878fcf566a19046	669	24
08992aedd5f963ec4c51688e4d980de5	616	2
6d23cba5806cb0cd964a510619774732	584	8
a557b6ca745a7236f67a7575098aad1e	578	2
0d5a9abf700cba2f3041046e57158f5d	574	10
481abe1c240a27677005da31d0d7a6c0	531	26
a39f3d8925ca6d49ab17fc8b1a333c1a	500	22
2808f5f8c95fb0018212221170abbddc	497	22
908b204b1242810169c973717e22e148	480	19
fcb419099fe77ad65f097cb4bc49a4a7	455	8
91d20ce368b224958b151ff03440ef98	451	9
5370ffbc2f778289e48d62501b271305	406	3
0218d72d766f03106b4db2198366e75a	386	23
3aacadafec0fc07fcbfdcf318ca07c9c	374	6
20690749005b354b43360bc864f35ab3	352	25
2a0cf99f0b2cfdf330e5f9408bc2d734	308	15
a07bc1b502bc0666b7e0f9ae35626391	289	7
5e0fb96dc99ad1e004c6467e9ccd2a52	272	19
aae9b9a6fb6d77234c967bf45dce5196	270	22
3d2404d3c57e42005c0a4658ae53f621	267	27
cfdd7f8a3be1419897613e2d161509ad	262	7
6e0e305a267f3644589dd48086a5c7cf	258	18
1ddf6bed8b8d2bf886a07e2078241e9e	242	22
c9b2ec35adcd43be7214c370052968fa	222	15
4d2d0f7bee11667ac420f44e1cb8b5f5	212	6
835c3e0ccf6a06b3ace58c9c6bae1fbd	212	6
cc231c20ce7de17921d5e22f37ee0fcc	201	22
cc5298164c630fb73fff1dd1714caa31	197	8
31dcb2b2171013304ef5ea5ad17e3e55	181	6
024fc7217da523a1c26d1a203a90d69e	177	9
9e6201146e8f882cb2efcd6ec66bdf5e	167	18
c3d4dc2a71f4e938cf14444eab2fb323	153	19
a1bc050e9ebf69966eaf6e4215f53353	143	4
66713f4eabc4899e97620b42c41863da	139	3
21ebdde4af3aea36188cb9eeb81eb154	137	4
376e72395ed71165c82e66b4716d831e	137	18
ee685de82a7ed29df93f98fcb171bd84	137	11
8bcc33c1e592d4673edcf002cb0eb3c9	132	9
1c02fbe4ea1fab6a1f4f65eeb3515d52	129	10

a90bf1263930109ee68c95c31c42ad24	121	10
3e21fc01f9867bb23868480f19006788	117	4
a0c56334474ad0fd891cd43bf6141b39	115	13
71eb09f1f6573b116eb4b89effa18e04	112	2
a9030113cda02ce233b9b23511dff2a7	105	8
6ed527434810e165be16c9c95c47c5d2	103	2
baf64ef771799a97062bd3bec4b0dab7	97	10
4479e6593cd34f2983abaf28bd59aee3	95	7
59ff8ddfbc86b1efa315c1e009cba249	94	8
f7c006c1694350e670d91f68e1697f8a	92	11
14a9ba70528cd8ad6c741bcf33bcb377	91	19
6c8d86a415057405c674b00bf950dee2	85	12
ae44199500a38ef76fe56332d6ba2d7f	83	12
d97b00eb1a99c4904dcf4bbfce422c60	83	1
2f9e8d691626abb8d898186c02abd5d0	80	7
d869071b67407df1d420d2a63aca204d	79	6
b52531c4c3f348cbf87603e83dc75664	73	2
f816e09e5a830b00f721c1f2d7f8e1e4	69	4
2214b091af4f2d0db950706ededb9aa1	69	5
cc71ec7192f81b0421f40c33862346b5	62	2
5285452f4c7157f7d83d547d37e41e8a	61	1
8c86684d1e18071691e6c92418ddbf02	60	2
72901e3c2682247cf755d7c69b38e5b9	54	2
2e13a15354eb43c0775fcd790894a461	50	8
98da7094bed979c123fd34abc082b3a7	50	2
968e041482eecf7c6ed0ad3e787b5d9b	48	4
7660db4fa0c5b5cd6a5a6dbb75fcf938	48	2
95ebcc80991eb72ffc0702e47b2ea9b3	48	7
e1d6be8ad9b33b0cf8c6670a58d4fe02	48	4
5a5e36deb9881b99643fa71055af0c37	46	1
0758c77edc17ccbf8b167aa6a15cb642	44	4
b22039c16c76dd1cda287a7e61ddf81b	41	2
181e18e15d37c16cafc4a46eceef6919	40	2
023dd9368380e822151f85f0f4ee5064	40	2
7201d37dcbd03148a6e97c8fbb81423f	39	6
51dead87dccaa7c310653c364dd76fae	39	3
2f09415dff8d1737f55e774fa5a84b8b	38	9
25bb3ebd83c6b1de0bba2fc90a2219f4	38	1
701b5245cc04ce7077d6b9ab549364b4	37	7
7d3e78ed3f515cc74304b0195e7a5e6e	35	1
056594fe74043a2671b1b00dbb42f4e2	35	2
19c250b38d235c3d05c4d77a534db8f8	35	3
6e6fe902082385517c8e293d7c38df21	34	1
fbd83ee3a227a84a8e2535c5205f4c59	34	1
8585dece87d47c8062cabe98ffe7980d	34	6
d5e68d412750a55cbb60e0dc87090e27	33	4
a2c412011174b2087698d5b57c83f715	32	4
c66b242a7611a3cf6b49be06e2eba70e	32	1
1d445488719a357864f8ffdaab1db55f	32	3
3731ca596da5a6367531831f34549891	31	1

1415df9554f154fe27e72c85276e16b3	30	2
f599a769f4c898cf494f7e76c35ed91c	30	1
4b87e805975efb43a7c78ff15b88a070	29	4
05d1122ee53417052ed32e92e58d309e	27	2
fcb774f7c472c785b6fdcb3845c4a9b6	27	1
2e185f30b2d24f4c8429474c78cca0c0	26	2
e4d030e1b629ba030897f21297afb47a	25	2
b079112f0d9727e63d3cb44bf79e3908	23	1
45b29c57b9f166e67685c5379120dbcf	23	1
4679f12460ee21e12953b3945325c198	23	4
c93d4e9a504c0bad8dcf1e228b0db316	22	1
b1001c9150aff0570d8e0259038b37f6	22	5
99e03611eb198d71cd951009235ef31a	21	2
074eeeecfdfedb0fb4d7da25c1e41fdf	21	1
7d7fabfb222636056d48d41d66e72c9f	20	2
03e699a77cf344b1ba5a0d40b4ce97ef	19	1
746564f133ddc97c0d83e56c8ad5c191	18	2
cc4304e1a0edb7bba4ef55a9f6bda63e	18	3
7b8a4fc45dc71da91d46d77f2524226e	18	2
932c3e2d662fb410bf81400423698a2a	17	2
7a201de781d4615f992a2fd876dbd375	17	2
a8d1e21961c013d5098d6a20c8983d83	17	1
8003cc28d7147a15777c5d5319f139fd	16	1
3655b6fbf4716a8728a85c2cd956c944	15	1
23e6778d3d7687ab4c7d876f6cbd8973	15	1
289e387c05a6d6fea33bf5a37a5290d7	15	3
f1bdb556a3a3d455c11d8167019d6a5e	15	1
6cd7a980df87acd750dbcbac54ab9c02	15	2
b0d75143194a87ff26ea477528d8ccb0	15	1
169bb33f9dd025e9771dd34b3e5f5f3d	14	1
2e460013f05fa603ba1c1de7031183b3	14	1
ac8e4b6b6d14ae61076c0232e7303be5	14	2
7ad2e778e5fb2a50c7747cbb5b3c1611	14	1
f7cdd302866822acdc419bde6db7243d	14	2
b194a57e5ce3d2c46f9e2d503f31fad9	13	1
39941e0c475b7077ea4a1ebdc6938bc6	13	1
a7f327109259259330d9fcbfdd72805f	13	1
5ec8bb2b4c344c4da8eacd3b24674157	13	1
e55e93306310b02fa0f24caa39c48c08	12	1
e6027d060759c2593d9813cf8a7121cd	12	4
2850ddb017e36aada207a5414bc2c359	11	1
cf6df8210fb3903769710267e1da9475	11	1
98f29adf8b23a3b7975c978b929268fb	10	2
d650ea974c038514fd6cea29ff9823e3	10	1
2c53067d70304de50cfbb8136fe1635e	10	1
1ac49d7edd5494969e82e42526aa1d0e	10	3
a3b4ec3e92126422273489a18317a012	10	1
47fd73b132f92012f43650a36f80caa9	10	3
7cea1277e914a269e74cf3e0d4297d05	10	1
fd35ee091a1df44c4cc0ac566689e383	9	1

6584de658d72346046f1c63d68cc9530	9	2
e4679ab100a1e3b4ad2f72c2936f9bb8	9	1
0c19336826fc10c8eac37262dc539622	9	2
588414bc3017040f3763d267982cd55b	9	1
9fe38673918f008bc46f33f87fb9da4c	8	2
42bee138c83cdda22dae6c8e31678f10	8	1
1ff115ee81637e65b411fa6f21b77f2f	8	1
b05a9780a4e7b87ba3a022887f928a60	8	1
5dabc57c7fba2ecaa7ea421de7684662	8	1
49c8b719d7aa7c856786903951b7d442	7	1
2ffea2b1f0e29444ce8955bbe8fedc7f	7	2
0192af45e9a0be54026a8a3d9adab3fd	7	1
6b9c27bffe95929531588aaf969fafff	7	1
e410046b700a620166ee3a172edc9564	7	2
a1693844150cbf95332f63d6e9a87eb3	7	1
4fbe7cc32e613d0434ad5f57c778bb4b	7	1
81b5c1b9e412668d1f551baf0cbfc725	7	1
42132176b300fc177390f22477e1969a	7	1
955462d88344f6d52e93c6f48816e319	7	1
3db3a4da3d40e7bd578055743a522563	6	1
1b024ce29ea8e7065cabb5b7e420f3a3	6	1
0241a01c836ad4a29e91f1fa381a2fbd	6	1
11e0cf997c0260d6ec7d4d5847f44b00	6	1
c0209e2acfdff2c26b8f047ea6c96dfa	6	1
2412bf0c2e092277e9c60a2bb6e7e4ba	6	1
93f3e9e09d63125cdc4d9aff9edd1d45	6	1
8fe210c40819bf1735c317e2bdc2d04e	6	1
e1eea436c00adbd0b7c2bfd082edd042	6	1
38894ed001e16d0aa285fd4d29044fea	6	1
ed2aa3491f9450fee20700baf790f53a	6	1
ecb4c0f87449cf04bb59b6e894edf9e2	6	1
f2af31f553d2812804234b832801cb1c	6	1
06b3eb7c4b11325f4670ee8bf797192c	5	1
fcf4b43bfcf6c51850324239e387e434	5	1
de4b18756403e43e2e37df4d2434f44e	5	1
238384ca735cb21dc3a3209a984f7de5	5	2
c8ad19f234b9f1646c44091b18c3df1c	5	1
750583592c67c9b27d28259ed862509b	5	1
16f55cc33aa90a334f06391e1e89eecf	5	1
6bbc0579297d23452e2625214fcb1eb0	5	1
b7fe2f51738ffba723c433db15fff699	5	1
59968d552533d76aae35d92bed666cac	5	2
0d023ed273812b0193ab0d4dfe0ddf99	5	1
b0a8e0bf37cbc759c9a3dcfb7ebdd339	5	1
854a549c2385492bcbe225c895e3db57	4	1
683df4e17f1f1d70012e495bd158a077	4	1
805a58a103f2546e9a257a772b163dc1	4	1
f5b1171ba916bb7ff6c0166f590aa85f	4	1
ee884167627591be4006729d60e46161	4	1
fc38de706763800bc6a93c8e6d373b72	4	1

86692a37af0ba511cf79c70f28732e95	4	1
7ff8313bd679520727ad9818ea14158b	4	1
63c9ba073dbabfa3f1199a2135ad91ec	4	1
3c0bea88efba1c8f3d84a1916f64300c	4	1
2514c3b40739ea02e9fa0de73867a018	4	1
087a83bdcc9ba60eaad132de29193a1e	3	1
64609bd25f8c73b17c24e0bc6999c18a	3	1
7cb298480601dafd20b6a5a64ec43368	3	1
c121eb4aeed68693639c6c97aa731ce7	3	1
3a01392f7a93ee628fee9d7bdf81e328	3	1
16b4fc34ffc7d658c3003bf71d262527	3	1
084c58b8b631b2d702d078f91f228c02	3	1
fc839a87d0c3d233535daf29c989baa6	3	1
4f252794952bf914a2165a589bcf48f9	3	1
76b9f9bd042761ee48a4fa8ac1ede5ca	3	1
370696bb190ade4fb1eaa6929800537e	3	1
194c1f6e6117b461faf8ed089bb45e2f	3	1
094f090854ef4c0813af139198f7fe1c	3	1
f7833fafd019341311ac8bb613ae47c7	3	1
c177aa74e66addffa5ff4e62ba311942	3	1
85cd518ae4e18c79a387593c8290fa76	3	1
ec18fd6241654ee5358da89f6efaa85d	2	1
02225615179dedc38fec61282f96c559	2	1
522348b5dc406bbf180c86ce158259f3	2	1
4f2d6ebf40c3a9d7c5c969b6d5491f3d	2	1
65c0d121b650be6412c538bb8581eaab	2	1
fd3f1d44f9767d5abaa084753ceb1f5f	2	1
99bfaf73cc4b797384e9dabc5f10342a	2	1
353a819a6d6451be3651768c0941a251	2	1
12da7e48fa3887f67f0325ab3bbee90f	2	1
99a685778b5da5c80378ff6d6eb600fd	2	1
8158341cfc498f9b40c942b449dc26bd	2	1
dbd3787d208606bf0cbe7e74de30a31b	2	1
44191952e54d6de1d65f76674dda8d45	2	1
648a222faa08620fa277a3eaabf6c253	1	1
cd80afbadb0797043698ca586260a017	1	1
3436d19e493a19ba6460574920b286e1	1	1
76a55a97dfc6db6fd19d5ac820a97495	1	1

TABLE S7 Table showing the frequencies of ASVs obtained from sequencing project for RNA (cDNA) samples from Run 4, 5 and 6.

ASV	Frequency	# of Samples Observed In
65496bbf4dc78382fac58935a32b1445	912.762	36
986967fd4264ba896aabed4c25f9a4af	446.139	36
1231c484790871f1b02b5b7352494fea	432.639	36
598d03e94e39c24ad9210b56ec86c67e	175.362	36
12e264fec25bbe1cc51d233b62ccc963	99.895	36
03713a8928ea62b4d6bf82d78a3b15a4	83.052	36
5436a782bc7ed40b8ad909655b0af7ce	81.437	36
e240646ffd33df766f41a7409825a044	70.695	36
179cbbc955a119e3c2353f48aa10bc5a	64.160	36
3663d41e5a13c3ac52ae7fbe31cfaa6b	56.519	36
61a40c5615f3cd435404098a5019af66	30.465	36
b66fc66e422a26a043680650483f7f8b	27.633	36
7db7c174c23363d553779ac968314491	24.852	36
97e0d6f1f616009df12e58179ef71fea	16.939	36
3bf9589bc24ee9c13d3f8e8a819011e4	16.185	36
b022b31257c230943cf27347f3dfa0c7	15.997	36
4373377f404bb9bf1f5b37c16662cd58	15.968	36
af3a07b12e3543eed55870c9056ffaf3	15.083	36
49c110de6638143ac4cc7ae844f93071	14.971	36
d99f15f84c339c746749b50a1cd1f71d	14.081	36
9e8a870c7a9c57f3d3b734318536d7f0	13.038	34
4541d7f9536c04dd1151a0be0ceee228	12.687	36
1deb11e859c78a082e2e11143f332e38	10.830	36
769cf91f760ccf78dfd1a4e0fbbb0b76	10.005	32
d51f208ff77a7e9eea303072812164f7	7.783	35
2332e8722d90259636448e1e82d9113e	7.756	36
d8f3c828aa33bec6cbd622058dd7e01a	7.190	34
22ca70d16470cad66451b56586898b92	6.932	36
ceacdcd5217a7d3be3456a2ce1da6a31	6.838	35
7fb1ca902a4d4a930b91c2492f409968	6.774	36
05a630bb34d7f103ca53692a43139459	5.536	34
ac623d76c0e59e6f53aee215a93c8497	5.021	36
4b347b76b57c2e71705057dd9dd1aed9	4.743	26
7624e20a982ba894a211695066600830	4.111	34
1c9d49371cb8032609739d5de05f50e5	3.969	23
91d20ce368b224958b151ff03440ef98	2.915	26
68146e68feac64537e12a9dd5a2a7e05	2.608	32
5df82dddb1aa7154e7783d76fbe1ce3d	2.189	34
e55bb4ef5c583951053e48c2ec554f3f	2.185	29
5829f592447e5b0d8784f43150ffb56f	2.100	29
a2d76b570fa0469135640884599a2af2	2.041	32
481abe1c240a27677005da31d0d7a6c0	1.835	36
5a71155f614b32e16173c607d01764a9	1.591	19
ad757d7046bd853d89f142d2c8466637	1.583	36
dd9e28ed02dfba5d507fadcf492d6713	1.520	23

58d0e3c2ce468949ea7570310c9cf17c	1.465	34
08992aedd5f963ec4c51688e4d980de5	1.420	2
33cd3dcab2602b1bb11a77c58850e841	1.358	33
625cea4e18659e393f3dfe3f3db04c3c	1.235	36
6c55ab68cd43b9974908c956a8a6ac9d	1.181	28
a557b6ca745a7236f67a7575098aad1e	1.144	2
cc231c20ce7de17921d5e22f37ee0fcc	814	34
6e0e305a267f3644589dd48086a5c7cf	798	24
35c8625cabb0f739a8bd5db7aed895a9	771	5
21ebdde4af3aea36188cb9eeb81eb154	699	14
6d23cba5806cb0cd964a510619774732	690	11
31dcb2b2171013304ef5ea5ad17e3e55	646	10
908b204b1242810169c973717e22e148	608	26
01591a6731e83bd7b04c607d8b6632eb	604	11
a07bc1b502bc0666b7e0f9ae35626391	569	7
20690749005b354b43360bc864f35ab3	511	30
a9030113cda02ce233b9b23511dff2a7	506	24
cc5298164c630fb73fff1dd1714caa31	434	6
fd5c4af273a2cddec97f31800dd1ea2b	371	22
cfdd7f8a3be1419897613e2d161509ad	344	7
2a0cf99f0b2cfdf330e5f9408bc2d734	331	12
05bb66f7bb196677ca067d5c19a424a4	314	21
54e82e478c7426290f25abfc59cd9556	301	8
9b028b89b8392d8f3d09fe92fb8f2b98	297	2
a39f3d8925ca6d49ab17fc8b1a333c1a	291	10
831a520fbfce915b3d2081449e9aa0a7	286	17
3e21fc01f9867bb23868480f19006788	285	8
19c250b38d235c3d05c4d77a534db8f8	278	5
a3da907655e9e9d2a32b1f1f11d530f6	263	2
71eb09f1f6573b116eb4b89effa18e04	252	2
9e6201146e8f882cb2efcd6ec66bdf5e	244	25
7201d37dcbd03148a6e97c8fbb81423f	230	11
ec486445d10300a4408e943c8a0709ed	229	1
3aacadafec0fc07fcbfdcf318ca07c9c	216	4
e121db2eb399a18856cfa05ca284c08f	214	5
4679f12460ee21e12953b3945325c198	214	11
5370ffbc2f778289e48d62501b271305	182	3
2808f5f8c95fb0018212221170abbddc	178	15
81a580e7ec333b555b97dba9f78e3246	168	1
4d2d0f7bee11667ac420f44e1cb8b5f5	168	5
024fc7217da523a1c26d1a203a90d69e	166	8
5e0fb96dc99ad1e004c6467e9ccd2a52	164	16
fcb419099fe77ad65f097cb4bc49a4a7	158	3
0758c77edc17ccbf8b167aa6a15cb642	141	6
bce3d14531e0e7da2878fcf566a19046	131	13
fcb774f7c472c785b6fdcb3845c4a9b6	126	4
cf799fb75c2c256239b2567d14f5e357	111	14
7b8a4fc45dc71da91d46d77f2524226e	109	13
376e72395ed71165c82e66b4716d831e	101	16
baf64ef771799a97062bd3bec4b0dab7	99	12

80e52039c3b6d220ee9b548770984f41	95	10
d7b9142fa18fa7e91bb0080e88fa02f5	92	15
34363eab7d41653ba9f4b878f109ab34	90	2
8bcc33c1e592d4673edcf002cb0eb3c9	89	8
3655b6fbf4716a8728a85c2cd956c944	88	4
86692a37af0ba511cf79c70f28732e95	79	13
5ffab526b6d78b4142b8d0da3f3ee025	74	15
a093b544a984f47aee992f07cacfeca8	72	2
51dead87dccaa7c310653c364dd76fae	70	3
023dd9368380e822151f85f0f4ee5064	65	2
8585dece87d47c8062cabe98ffe7980d	60	8
19bb4b1d639a45b8d6483ef7625508cd	59	7
c664afbac1b5d466fe81bb9d13b702f8	57	1
e6027d060759c2593d9813cf8a7121cd	55	7
d869071b67407df1d420d2a63aca204d	52	7
9fe38673918f008bc46f33f87fb9da4c	50	4
181e18e15d37c16cafc4a46eceef6919	49	4
a1bc050e9ebf69966eaf6e4215f53353	49	2
835c3e0ccf6a06b3ace58c9c6bae1fbd	46	5
3731ca596da5a6367531831f34549891	44	2
e1296138e81340b8696320b6e62108c2	43	3
77ac02c2d6e75ac848fd96f113337ca0	42	3
5a3b46b65391e2c10eedc6d3eb982816	40	1
7f55b63cb38020db11785af2393472d9	37	2
366c2d7c522e56c3c16d12d02925d240	33	3
1c02fbe4ea1fab6a1f4f65eeb3515d52	33	2
cc71ec7192f81b0421f40c33862346b5	33	3
581ffe5fe7e2e6f54e192cd84ec0eb03	32	2
3db3a4da3d40e7bd578055743a522563	31	1
f2e1c13f7f01a9285643e64067afb4da	30	1
c3d4dc2a71f4e938cf14444eab2fb323	28	9
38eb886566b99ab196c4e435c131642d	28	3
aae9b9a6fb6d77234c967bf45dce5196	28	5
43fed92fc45d2b5155e286723d0053bb	27	1
c9b2ec35adcd43be7214c370052968fa	26	3
129f69b05258d4a91e474d7e04f5e7ad	26	1
2d158e47f5cf402e1b9149354823ce64	25	1
c66b242a7611a3cf6b49be06e2eba70e	25	1
5523f2502c26de41d8f60595f7d13ee5	24	1
9056a14ab8d483816142f9552d088377	24	1
ae44199500a38ef76fe56332d6ba2d7f	23	4
a7f327109259259330d9fcbfdd72805f	22	2
561ec58852a6e0f85a38e67ea8f531c5	21	1
6e68eb169373ddcf53e32f5ee333ef34	20	1
402aa791075837702ec3a303814c53e4	20	1
968e041482eecf7c6ed0ad3e787b5d9b	19	2
5b3f8c0cc0cce1421ab5c105310b34b1	18	1
7660db4fa0c5b5cd6a5a6dbb75fcf938	18	1
cc4304e1a0edb7bba4ef55a9f6bda63e	17	3
9925bea90bbc9b7a2c5f0bc33018e58e	16	1

a87ae72f7bb392c83aa6c67a4317f7e5	16	1
4e03df8922f5c72d254c807cb0bcb20d	16	2
1415df9554f154fe27e72c85276e16b3	15	1
169bb33f9dd025e9771dd34b3e5f5f3d	15	2
98f29adf8b23a3b7975c978b929268fb	15	1
b194a57e5ce3d2c46f9e2d503f31fad9	14	1
2e460013f05fa603ba1c1de7031183b3	14	1
f1de4e20743453d7657ae591aa5912a3	14	1
1ac49d7edd5494969e82e42526aa1d0e	14	2
c3ba165e798fc36af3715c360db594fd	13	2
c959802818a388b43afb95d9f17a58fa	13	1
1b4b73ca73a28ac494503a1b1daad14a	11	1
c8549a8d5a41c9dc8b7093b730331a3b	11	1
81c20363b1773bc3bb4ddcd0e7720d88	11	1
7261200c3b63695ba38e18cdc25f016a	11	1
ed1cec48a8107df03ba29afadf090049	11	1
ed1db9375ec6f1a673eeeea432ecb012	11	1
6e43a247827ff3c947e211ff54bfa39a	10	1
67c4972a059ce750fe3e14629cda5290	10	1
b383b586fe3874fb768767d3b7ef8b42	10	1
aef4b2637d507f6f75a200c2f5bb1658	10	1
79437884578b9d4fc2370f27a87fce75	9	1
66e2b1a8ac28a283c592ee5bf0a8b482	9	1
c07d88d9e8e228ae89ccd09fd465a44a	9	1
6593ba4d1e51c31ef13d87c5016f4a32	9	3
1f48d06fde0de8a1805385cf2c1ec529	8	1
4fbe7cc32e613d0434ad5f57c778bb4b	8	1
93f3e9e09d63125cdc4d9aff9edd1d45	8	2
254ea9214bf8a7903747f1bc023d3aa5	8	1
8304c0dc3ce08f43e5eb5ce31099ba80	7	1
084c58b8b631b2d702d078f91f228c02	7	2
16f55cc33aa90a334f06391e1e89eecf	7	1
f816e09e5a830b00f721c1f2d7f8e1e4	7	1
0293bf9f70cf9100d0a887ff6980749f	7	1
821b2d992c23efab3a16b43573ef0ab4	7	1
6f17552b4da678ee1544bbaebda5ebcf	7	1
8c20f1408895276c6ca9f58d852df30b	7	1
4ac7d3006cd4da603a5d3cab61249f7f	6	1
f00ccc22da3dee27522f7f2ae50b55cf	6	1
14a9ba70528cd8ad6c741bcf33bcb377	6	1
d06865e3c82f698c6d7c0d5a8baef6e7	6	1
51aa28b0a62986428aa6359bdcd2a83f	6	1
cad50aafa44beed19f9f12776ed0551d	6	1
65c0d121b650be6412c538bb8581eaab	6	1
4615c33bd0612615823860f432de266a	5	1
1d445488719a357864f8ffdaab1db55f	5	1
d2549689ff457ae53646f6fc86383d79	5	1
ed4bb998edf68b609e0b58375096ccfd	5	1
4025ce7628c913379c32e51ec313c295	5	1
1ddf6bed8b8d2bf886a07e2078241e9e	5	1

ee884167627591be4006729d60e46161	5	2
3ca4389c31d3d83c73460dbc12568704	5	1
dbe034d7bd68c5191c21d34cc24f66f4	5	1
59ff8ddfbc86b1efa315c1e009cba249	4	1
43f44d35dcb40b558abed7433cd0d6c1	4	1
ca8286e24702cc1b2d29646bccd0f240	4	1
0218d72d766f03106b4db2198366e75a	4	1
85cd518ae4e18c79a387593c8290fa76	4	1
4a2206c810686389982b688de795d5a3	4	1
5717b96bbe0946e2209b0e14e4f828d3	4	1
121e8516a8415e3e0009b2cdedb2dfbe	4	1
797ec82b253d67346dc90592d8817fed	3	1
e577a127861784ad863b076a328025c7	3	1
6c8d86a415057405c674b00bf950dee2	3	1
5f9b1a144910192fad1bd4a3d8b8b58c	3	1
9a8fe698727de87bbb2eb1e40e51e4d3	3	1
9c06e714c37f27a159af8b67686d8139	3	1
ef66ec977ae8df1fd6bd734fec4c8117	3	1
a318d0193883d429e0199320aa6f4d64	3	1
b45f4bf35843cc2c1c61488fbc51ebf9	3	1
a0c56334474ad0fd891cd43bf6141b39	2	1
5290c49da4492a10edd7988865edace6	2	1
56e30419dedfd2d957e6ca571ed36d2e	2	1
238384ca735cb21dc3a3209a984f7de5	2	1
e1eea436c00adbd0b7c2bfd082edd042	2	1
062a6977877aacfef233a53cd4ae6a7f	2	1
675aa026dcd120f995ea7e16285f6e90	2	1
62b3dfe4921ac5d5359ac0af96cbd333	2	1
aca79e16df499731584dc67f4fa9f4a7	2	1
eb9613cf8dc457220dad25313db1ab53	2	1
fb9eb3e4fa2b707587fe404377f55e00	2	1
b459b4bf78b5a5e203775d99e9302b3d	1	1
214e56e9f1178d6f4d1fbbc8631d7fb5	1	1
f92702d401a3873c6417e7a698b95e4b	1	1
701b5245cc04ce7077d6b9ab549364b4	1	1
ba6cf4bc54758d2a3ef686d91b6d6a57	1	1
a1ce8303774e21bdaaa2bc8acfb60f90	1	1
b71d72336f500d0d08583d03fc52909f	1	1
6181fe556c5a503eb2b68d2176cd6d94	1	1
569bcc1c0f806fd9c7f7e47b87c66dc1	1	1
3bdc5a64cf58117685d64cc75f890377	1	1
cacfc0210433ebc36e55d1cf50bfefc9	1	1
fa742c6f0fe519acd2e64322d000cc94	1	1
8b1989d79ea5cc5e6aac800b1bc3b3d2	1	1
087a83bdcc9ba60eaad132de29193a1e	1	1

TABLE S8 Table showing the frequencies of ASVs obtained after merging of ASVs from all sequencing projects for both DNA and RNA

ASV	Frequency	# of Samples Observed In
986967fd4264ba896aabed4c25f9a4af	1.613.524	112
65496bbf4dc78382fac58935a32b1445	1.608.245	112
598d03e94e39c24ad9210b56ec86c67e	1.162.377	112
1231c484790871f1b02b5b7352494fea	1.137.776	112
5436a782bc7ed40b8ad909655b0af7ce	531.807	112
7db7c174c23363d553779ac968314491	199.405	112
12e264fec25bbe1cc51d233b62ccc963	157.235	81
179cbbc955a119e3c2353f48aa10bc5a	152.983	112
03713a8928ea62b4d6bf82d78a3b15a4	149.974	112
e240646ffd33df766f41a7409825a044	121.117	112
b66fc66e422a26a043680650483f7f8b	84.838	112
3663d41e5a13c3ac52ae7fbe31cfaa6b	68.974	112
4373377f404bb9bf1f5b37c16662cd58	56.862	112
d99f15f84c339c746749b50a1cd1f71d	51.310	112
61a40c5615f3cd435404098a5019af66	51.047	111
97e0d6f1f616009df12e58179ef71fea	50.856	112
af3a07b12e3543eed55870c9056ffaf3	49.499	112
49c110de6638143ac4cc7ae844f93071	42.660	112
b022b31257c230943cf27347f3dfa0c7	41.257	112
1deb11e859c78a082e2e11143f332e38	32.868	112
9e8a870c7a9c57f3d3b734318536d7f0	23.833	92
4541d7f9536c04dd1151a0be0ceee228	23.039	92
ceacdcd5217a7d3be3456a2ce1da6a31	22.150	110
22ca70d16470cad66451b56586898b92	21.063	112
d8f3c828aa33bec6cbd622058dd7e01a	20.359	97
7fb1ca902a4d4a930b91c2492f409968	20.077	112
d51f208ff77a7e9eea303072812164f7	19.837	96
3bf9589bc24ee9c13d3f8e8a819011e4	18.667	64
ac623d76c0e59e6f53aee215a93c8497	13.248	111
58d0e3c2ce468949ea7570310c9cf17c	12.562	110
769cf91f760ccf78dfd1a4e0fbbb0b76	12.050	45
2332e8722d90259636448e1e82d9113e	10.552	71
e5046e5c557d1f90925a8ed986a0ef29	10.293	48
5829f592447e5b0d8784f43150ffb56f	10.268	56
625cea4e18659e393f3dfe3f3db04c3c	10.261	112
05bb66f7bb196677ca067d5c19a424a4	10.066	96
5df82dddb1aa7154e7783d76fbe1ce3d	9.034	109
dd9e28ed02dfba5d507fadcf492d6713	8.743	77
4b347b76b57c2e71705057dd9dd1aed9	8.508	49
e55bb4ef5c583951053e48c2ec554f3f	8.376	103
5a71155f614b32e16173c607d01764a9	8.125	69
83445197c16bee473991239f7e15f11c	8.096	48
68146e68feac64537e12a9dd5a2a7e05	7.607	93
7624e20a982ba894a211695066600830	6.234	89

a2d76b570fa0469135640884599a2af2	6.138	106
1c9d49371cb8032609739d5de05f50e5	5.901	34
05a630bb34d7f103ca53692a43139459	5.536	34
33cd3dcab2602b1bb11a77c58850e841	5.393	107
fcb419099fe77ad65f097cb4bc49a4a7	5.326	52
831a520fbfce915b3d2081449e9aa0a7	4.746	71
ad757d7046bd853d89f142d2c8466637	4.484	112
481abe1c240a27677005da31d0d7a6c0	4.465	105
91d20ce368b224958b151ff03440ef98	3.636	57
6c55ab68cd43b9974908c956a8a6ac9d	3.631	97
9ed27f4bddcb1afb25681e918566a55d	3.409	20
01591a6731e83bd7b04c607d8b6632eb	2.964	40
77ac02c2d6e75ac848fd96f113337ca0	2.548	39
cc231c20ce7de17921d5e22f37ee0fcc	2.507	103
6d23cba5806cb0cd964a510619774732	2.311	28
9b028b89b8392d8f3d09fe92fb8f2b98	2.167	17
08992aedd5f963ec4c51688e4d980de5	2.036	4
835c3e0ccf6a06b3ace58c9c6bae1fbd	1.912	44
80e52039c3b6d220ee9b548770984f41	1.899	34
a557b6ca745a7236f67a7575098aad1e	1.722	4
6297cf382b94ea9f464a72a995fea3c0	1.485	25
bce3d14531e0e7da2878fcf566a19046	1.473	63
20690749005b354b43360bc864f35ab3	1.461	89
a39f3d8925ca6d49ab17fc8b1a333c1a	1.375	55
908b204b1242810169c973717e22e148	1.352	64
fd5c4af273a2cddec97f31800dd1ea2b	1.310	64
8bcc33c1e592d4673edcf002cb0eb3c9	1.265	52
34363eab7d41653ba9f4b878f109ab34	1.261	17
6e0e305a267f3644589dd48086a5c7cf	1.213	59
16180afbe4300d9862ebcc81f3914397	1.178	7
2808f5f8c95fb0018212221170abbddc	1.110	58
21ebdde4af3aea36188cb9eeb81eb154	1.019	22
a07bc1b502bc0666b7e0f9ae35626391	971	17
a9030113cda02ce233b9b23511dff2a7	872	48
31dcb2b2171013304ef5ea5ad17e3e55	868	19
cc3d5fb92638a2314ef5c5e3dd9d34b7	790	14
35c8625cabb0f739a8bd5db7aed895a9	771	5
5370ffbc2f778289e48d62501b271305	754	8
1dadaaefef4d77778b805052d119f82a	742	11
c707860eb7d75197d4bffc989187fe71	722	25
3e21fc01f9867bb23868480f19006788	711	23
0d5a9abf700cba2f3041046e57158f5d	682	17
2a0cf99f0b2cfdf330e5f9408bc2d734	679	29
cc5298164c630fb73fff1dd1714caa31	677	18
fcacbc2a90b3e158762926d9ab3178ec	654	12
0218d72d766f03106b4db2198366e75a	647	38
238384ca735cb21dc3a3209a984f7de5	630	29
19c250b38d235c3d05c4d77a534db8f8	623	14
cfdd7f8a3be1419897613e2d161509ad	606	14
4fc6daa94b75fb73fe80656de5730631	596	11

3aacadafec0fc07fcbfdcf318ca07c9c	590	10
a1bc050e9ebf69966eaf6e4215f53353	534	25
efc4bb5dc12fcef552cfaa5d4ea1cb61	527	22
5e0fb96dc99ad1e004c6467e9ccd2a52	494	46
9e6201146e8f882cb2efcd6ec66bdf5e	477	50
c3d4dc2a71f4e938cf14444eab2fb323	465	53
3d2404d3c57e42005c0a4658ae53f621	446	47
4d2d0f7bee11667ac420f44e1cb8b5f5	426	14
e121db2eb399a18856cfa05ca284c08f	405	10
1a87be1b3fd874307d6334aa57e432f6	369	15
71eb09f1f6573b116eb4b89effa18e04	364	4
024fc7217da523a1c26d1a203a90d69e	343	17
1ddf6bed8b8d2bf886a07e2078241e9e	336	33
aae9b9a6fb6d77234c967bf45dce5196	336	30
c9b2ec35adcd43be7214c370052968fa	304	25
6c5a6031a86bcd5b3e9e0e7333112c39	301	6
54e82e478c7426290f25abfc59cd9556	301	8
7201d37dcbd03148a6e97c8fbb81423f	285	20
376e72395ed71165c82e66b4716d831e	275	39
a3da907655e9e9d2a32b1f1f11d530f6	263	2
ee685de82a7ed29df93f98fcb171bd84	239	19
4679f12460ee21e12953b3945325c198	237	15
ec486445d10300a4408e943c8a0709ed	229	1
1c02fbe4ea1fab6a1f4f65eeb3515d52	226	17
6c8d86a415057405c674b00bf950dee2	197	24
baf64ef771799a97062bd3bec4b0dab7	196	22
0758c77edc17ccbf8b167aa6a15cb642	185	10
19bb4b1d639a45b8d6483ef7625508cd	182	13
66713f4eabc4899e97620b42c41863da	170	7
81a580e7ec333b555b97dba9f78e3246	168	1
3982b3368d72d1c51207dfbb6cbe24d8	161	3
fcb774f7c472c785b6fdcb3845c4a9b6	153	5
a90bf1263930109ee68c95c31c42ad24	147	14
cf799fb75c2c256239b2567d14f5e357	143	19
86692a37af0ba511cf79c70f28732e95	140	21
181e18e15d37c16cafc4a46eceef6919	139	9
cc71ec7192f81b0421f40c33862346b5	137	7
6ed527434810e165be16c9c95c47c5d2	137	3
8585dece87d47c8062cabe98ffe7980d	137	15
14a9ba70528cd8ad6c741bcf33bcb377	137	25
7b8a4fc45dc71da91d46d77f2524226e	131	16
d869071b67407df1d420d2a63aca204d	131	13
ae44199500a38ef76fe56332d6ba2d7f	122	19
51dead87dccaa7c310653c364dd76fae	120	8
a4761f7c93d515df40deab14b48777bf	119	5
a0c56334474ad0fd891cd43bf6141b39	117	14
85cd518ae4e18c79a387593c8290fa76	117	15
4479e6593cd34f2983abaf28bd59aee3	106	9
e6027d060759c2593d9813cf8a7121cd	106	18
023dd9368380e822151f85f0f4ee5064	105	4

3655b6fbf4716a8728a85c2cd956c944	103	5
57c34d52c453f938ab74b0cb3aab9583	103	5
59ff8ddfbc86b1efa315c1e009cba249	98	9
f7c006c1694350e670d91f68e1697f8a	98	12
d7b9142fa18fa7e91bb0080e88fa02f5	92	15
d97b00eb1a99c4904dcf4bbfce422c60	83	1
2f09415dff8d1737f55e774fa5a84b8b	83	17
2f9e8d691626abb8d898186c02abd5d0	80	7
b52531c4c3f348cbf87603e83dc75664	79	3
968e041482eecf7c6ed0ad3e787b5d9b	78	8
5ffab526b6d78b4142b8d0da3f3ee025	77	17
f816e09e5a830b00f721c1f2d7f8e1e4	76	5
3731ca596da5a6367531831f34549891	75	3
a093b544a984f47aee992f07cacfeca8	72	2
7a201de781d4615f992a2fd876dbd375	72	6
5bea5cfdb8bff735befd718017ea1a8c	70	4
2214b091af4f2d0db950706ededb9aa1	69	5
23e6778d3d7687ab4c7d876f6cbd8973	66	4
7660db4fa0c5b5cd6a5a6dbb75fcf938	66	3
98da7094bed979c123fd34abc082b3a7	65	3
2e13a15354eb43c0775fcd790894a461	64	11
2ffea2b1f0e29444ce8955bbe8fedc7f	63	5
f57e6a8686e524991a4da35eaedb5c7a	63	1
8c86684d1e18071691e6c92418ddbf02	63	3
ceed4dc4dddcce320ea6b073db284644	62	4
5285452f4c7157f7d83d547d37e41e8a	61	1
9fe38673918f008bc46f33f87fb9da4c	58	6
c664afbac1b5d466fe81bb9d13b702f8	57	1
c66b242a7611a3cf6b49be06e2eba70e	57	2
e1d6be8ad9b33b0cf8c6670a58d4fe02	57	5
0335a90f3710821afb3afaf9e3167868	54	7
72901e3c2682247cf755d7c69b38e5b9	54	2
7f55b63cb38020db11785af2393472d9	53	4
05d1122ee53417052ed32e92e58d309e	52	4
45b29c57b9f166e67685c5379120dbcf	50	3
812e739c966ac77def6a2623efc0b848	49	1
95ebcc80991eb72ffc0702e47b2ea9b3	48	7
f599a769f4c898cf494f7e76c35ed91c	46	2
5a5e36deb9881b99643fa71055af0c37	46	1
1415df9554f154fe27e72c85276e16b3	45	3
7cea1277e914a269e74cf3e0d4297d05	45	5
9ffb8551e2a8eda9ec80d31fb0ede081	44	1
20cf65e815e5dbb356c800fc92f03676	44	1
701b5245cc04ce7077d6b9ab549364b4	44	9
e1296138e81340b8696320b6e62108c2	43	3
b22039c16c76dd1cda287a7e61ddf81b	41	2
d5e68d412750a55cbb60e0dc87090e27	41	6
5a3b46b65391e2c10eedc6d3eb982816	40	1
25bb3ebd83c6b1de0bba2fc90a2219f4	38	1
1d445488719a357864f8ffdaab1db55f	37	4

03e699a77cf344b1ba5a0d40b4ce97ef	37	2
3db3a4da3d40e7bd578055743a522563	37	2
99e03611eb198d71cd951009235ef31a	36	4
a7f327109259259330d9fcbfdd72805f	35	3
7d3e78ed3f515cc74304b0195e7a5e6e	35	1
cc4304e1a0edb7bba4ef55a9f6bda63e	35	6
0c19336826fc10c8eac37262dc539622	35	4
056594fe74043a2671b1b00dbb42f4e2	35	2
fbd83ee3a227a84a8e2535c5205f4c59	34	1
6e6fe902082385517c8e293d7c38df21	34	1
9056a14ab8d483816142f9552d088377	34	2
366c2d7c522e56c3c16d12d02925d240	33	3
efc1610ee23b8fd2b868d52265f7ba75	32	2
c121eb4aeed68693639c6c97aa731ce7	32	6
a2c412011174b2087698d5b57c83f715	32	4
581ffe5fe7e2e6f54e192cd84ec0eb03	32	2
1ac49d7edd5494969e82e42526aa1d0e	30	6
00a330dbadc40c961ed97fb3d88897bc	30	1
f2e1c13f7f01a9285643e64067afb4da	30	1
169bb33f9dd025e9771dd34b3e5f5f3d	29	3
b4856372de34260b5e8f83360eb40b4f	29	2
4b87e805975efb43a7c78ff15b88a070	29	4
ea1769adc4181375706eecd97394b8c0	29	5
2e460013f05fa603ba1c1de7031183b3	28	2
38eb886566b99ab196c4e435c131642d	28	3
43fed92fc45d2b5155e286723d0053bb	27	1
b194a57e5ce3d2c46f9e2d503f31fad9	27	2
129f69b05258d4a91e474d7e04f5e7ad	26	1
2e185f30b2d24f4c8429474c78cca0c0	26	2
e4d030e1b629ba030897f21297afb47a	25	2
4ac7d3006cd4da603a5d3cab61249f7f	25	2
98f29adf8b23a3b7975c978b929268fb	25	3
2d158e47f5cf402e1b9149354823ce64	25	1
ab39860d7aad8f55ff2e37cf4679cc1e	25	1
5523f2502c26de41d8f60595f7d13ee5	24	1
faf23e14bc07d8c09491f3361f71d807	23	1
b079112f0d9727e63d3cb44bf79e3908	23	1
c93d4e9a504c0bad8dcf1e228b0db316	22	1
b1001c9150aff0570d8e0259038b37f6	22	5
1f48d06fde0de8a1805385cf2c1ec529	22	2
eb9a53bb5e4baa670f0c3b3f960deebb	22	4
074eeeecfdfedb0fb4d7da25c1e41fdf	21	1
561ec58852a6e0f85a38e67ea8f531c5	21	1
3bfefdea4f56503bb045813dc77a5179	21	1
42bee138c83cdda22dae6c8e31678f10	21	2
946c3853f952f96ab2811a0c046462d5	21	1
6e68eb169373ddcf53e32f5ee333ef34	20	1
02bfbf94e7a6e52f2ff78a7b09a86d83	20	1
402aa791075837702ec3a303814c53e4	20	1
7d7fabfb222636056d48d41d66e72c9f	20	2

2d0318b452ca34384b89875743824048	20	1
2c53067d70304de50cfbb8136fe1635e	20	3
9925bea90bbc9b7a2c5f0bc33018e58e	19	2
1b4b73ca73a28ac494503a1b1daad14a	19	2
12b85924c194c76cfa9c7d89ce9376e5	18	1
da003c5b80bcce6c7867a28ac3b2e9e6	18	4
5b3f8c0cc0cce1421ab5c105310b34b1	18	1
6c18638076676fa55fd3a4f8c3449f56	18	1
5d4bd876d644427e8865fcfe49c7fabc	18	2
fb91cdba406d4a0b4ff9da4c2872ff31	18	1
746564f133ddc97c0d83e56c8ad5c191	18	2
7515e6d25268ec6b1cf71862c597e663	17	1
a8d1e21961c013d5098d6a20c8983d83	17	1
932c3e2d662fb410bf81400423698a2a	17	2
a113d5803b9ab120ed8651984f0fee72	16	1
8003cc28d7147a15777c5d5319f139fd	16	1
3f9e45056e4bff201076d5da60d19141	16	1
9bcf746120a35ec999eebf145e168e30	16	1
a87ae72f7bb392c83aa6c67a4317f7e5	16	1
4e03df8922f5c72d254c807cb0bcb20d	16	2
6cd7a980df87acd750dbcbac54ab9c02	15	2
b0d75143194a87ff26ea477528d8ccb0	15	1
4fbe7cc32e613d0434ad5f57c778bb4b	15	2
f1bdb556a3a3d455c11d8167019d6a5e	15	1
2161fd52308152215dcdec19702907d5	15	1
289e387c05a6d6fea33bf5a37a5290d7	15	3
6593ba4d1e51c31ef13d87c5016f4a32	15	4
994db2eb36da4b53de581daf4ad9e2dc	15	1
63027a8f109a3a591fe6c6dae22b10cb	15	3
fd35ee091a1df44c4cc0ac566689e383	15	2
f1de4e20743453d7657ae591aa5912a3	14	1
f7cdd302866822acdc419bde6db7243d	14	2
b439f154aca81bfde353580eb801ee4e	14	1
86b091d4fc7f9a87735339bb72502026	14	2
93f3e9e09d63125cdc4d9aff9edd1d45	14	3
e4fdb08c6bdb194ee9bd4a80de51ce0f	14	1
ac8e4b6b6d14ae61076c0232e7303be5	14	2
7ad2e778e5fb2a50c7747cbb5b3c1611	14	1
a421ba0dcd3ce10ece9a8f495adb3faf	14	1
4f2d6ebf40c3a9d7c5c969b6d5491f3d	13	3
5ec8bb2b4c344c4da8eacd3b24674157	13	1
c4677339dd0a745c23597cc60f29ea24	13	1
c959802818a388b43afb95d9f17a58fa	13	1
4d07c4262b7e7e40dedd88d23e7f224e	13	2
c3ba165e798fc36af3715c360db594fd	13	2
39941e0c475b7077ea4a1ebdc6938bc6	13	1
16f55cc33aa90a334f06391e1e89eecf	12	2
905ef8101f26383dfeb299eef0f7b6f9	12	1
e55e93306310b02fa0f24caa39c48c08	12	1
1666bcd414a0fcd8881a4fb5ba9ff702	12	1

57bb4e11927bb0dd9b48ec8fa0230205	11	1
ed1cec48a8107df03ba29afadf090049	11	1
a871e00cb5c460c97704ea57519c52e9	11	1
ed1db9375ec6f1a673eeeea432ecb012	11	1
2850ddb017e36aada207a5414bc2c359	11	1
331e47bc95931574576e67e0b4738697	11	1
2c3d88d72198fa47b8f8c9270cc56485	11	1
4fc4b46df175d368659d622d8288ed16	11	1
df635d0ebc5626f0d25c492d875c65ef	11	1
e410046b700a620166ee3a172edc9564	11	3
81c20363b1773bc3bb4ddcd0e7720d88	11	1
d14ef7597f581525ec2607e6b591f12a	11	1
cf6df8210fb3903769710267e1da9475	11	1
7261200c3b63695ba38e18cdc25f016a	11	1
c8549a8d5a41c9dc8b7093b730331a3b	11	1
04f42921bdf8c6ba78d3dc9d08dbaff6	11	1
084c58b8b631b2d702d078f91f228c02	10	3
47fd73b132f92012f43650a36f80caa9	10	3
b383b586fe3874fb768767d3b7ef8b42	10	1
0fe676474cb62e5781a215eeb66b895a	10	1
a3b4ec3e92126422273489a18317a012	10	1
d650ea974c038514fd6cea29ff9823e3	10	1
67c4972a059ce750fe3e14629cda5290	10	1
aef4b2637d507f6f75a200c2f5bb1658	10	1
6e43a247827ff3c947e211ff54bfa39a	10	1
ee884167627591be4006729d60e46161	9	3
42f7e686e76739283cbb4fef7a570aa0	9	1
e4679ab100a1e3b4ad2f72c2936f9bb8	9	1
c59a118afdc652913766de4b1753f102	9	1
588414bc3017040f3763d267982cd55b	9	1
12dc019695ba2d4d8f648eef6b0d3896	9	1
e3195d003899773618689ae607fd7b99	9	1
a22ce57580bc2220580ebf9909410777	9	1
c07d88d9e8e228ae89ccd09fd465a44a	9	1
b1299a1ca4669ebf91e7d4053be86b35	9	1
1e22e775a1ac07f39ba599d6683b4e2f	9	1
66e2b1a8ac28a283c592ee5bf0a8b482	9	1
6584de658d72346046f1c63d68cc9530	9	2
79437884578b9d4fc2370f27a87fce75	9	1
b05a9780a4e7b87ba3a022887f928a60	8	1
1ff115ee81637e65b411fa6f21b77f2f	8	1
eab937ecd354d0c94fc1f94db4c0e1ba	8	1
e7ff57b2d8a75972c5a74082f9fea66d	8	1
254ea9214bf8a7903747f1bc023d3aa5	8	1
4bdffa859d3f9098f6fa14ee1afc96e5	8	1
d70c5d0b986bf2447fed1cb9a542da18	8	1
65c0d121b650be6412c538bb8581eaab	8	2
19d1e2652e6ebff7e8ca2b4ab2942548	8	1
e32f54287d83be049632e61f9e6fb091	8	1
e1eea436c00adbd0b7c2bfd082edd042	8	2

5dabc57c7fba2ecaa7ea421de7684662	8	1
821b2d992c23efab3a16b43573ef0ab4	7	1
81b5c1b9e412668d1f551baf0cbfc725	7	1
c4c424c3c7da1c004ded51bf948f8900	7	1
0293bf9f70cf9100d0a887ff6980749f	7	1
f7f2737b8e1459c317ffa3c4ebe126bc	7	1
49c8b719d7aa7c856786903951b7d442	7	1
8304c0dc3ce08f43e5eb5ce31099ba80	7	1
9f1e537ae1b81eba9129f16da13f8fe0	7	1
3184b5d04dfdb737d5ecaea90f070c9d	7	2
38a43e4aab5a8b8b44fa19f441707e15	7	1
6b9c27bffe95929531588aaf969fafff	7	1
8c20f1408895276c6ca9f58d852df30b	7	1
a1693844150cbf95332f63d6e9a87eb3	7	1
955462d88344f6d52e93c6f48816e319	7	1
42132176b300fc177390f22477e1969a	7	1
6f17552b4da678ee1544bbaebda5ebcf	7	1
4dd9f814152f6b2d917ee64f6d7880d8	7	1
0192af45e9a0be54026a8a3d9adab3fd	7	1
0a2ada1ea7f4be8361ccea4dbbf40777	7	2
87a9d09285ca978c6c6bade91d308af9	7	1
260e85089fc5e09c2e31f03d458b4cb0	7	1
7fb24aa3bf5c45149195831a5b84ed17	7	1
05bfcca37a7eed3ede4a1d804d33f8f6	7	1
f2af31f553d2812804234b832801cb1c	6	1
f00ccc22da3dee27522f7f2ae50b55cf	6	1
675aa026dcd120f995ea7e16285f6e90	6	2
235489e885312c5a8524ea48fa4252e2	6	1
2412bf0c2e092277e9c60a2bb6e7e4ba	6	1
30b31fb66650424d81a9bd6f60224000	6	1
d7dbe417ddb2087201bd9549ea0eccdc	6	1
9e137da4097f161ff4648760463590dc	6	1
d06865e3c82f698c6d7c0d5a8baef6e7	6	1
02b3f5a0f26f23a214129736106baac1	6	1
cad50aafa44beed19f9f12776ed0551d	6	1
0241a01c836ad4a29e91f1fa381a2fbd	6	1
38894ed001e16d0aa285fd4d29044fea	6	1
8fe210c40819bf1735c317e2bdc2d04e	6	1
51aa28b0a62986428aa6359bdcd2a83f	6	1
1b024ce29ea8e7065cabb5b7e420f3a3	6	1
ecb4c0f87449cf04bb59b6e894edf9e2	6	1
ed2aa3491f9450fee20700baf790f53a	6	1
11e0cf997c0260d6ec7d4d5847f44b00	6	1
4954c6218cb7f3d9160d63d24a464dc1	6	1
c0209e2acfdff2c26b8f047ea6c96dfa	6	1
a4a5656e4cf33523cec6b4b495d5082b	5	1
fcf4b43bfcf6c51850324239e387e434	5	1
b7fe2f51738ffba723c433db15fff699	5	1
750583592c67c9b27d28259ed862509b	5	1
59968d552533d76aae35d92bed666cac	5	2

b0a8e0bf37cbc759c9a3dcfb7ebdd339	5	1
de4b18756403e43e2e37df4d2434f44e	5	1
4c0191d947cbf14d60855ae5de0d3ac8	5	1
14caed348f1f188683986fc601c34c7c	5	1
fe68b7957842ec0d4d3ca4bdaf4fe471	5	1
c8ad19f234b9f1646c44091b18c3df1c	5	1
08e4f7408eb3821919acab4dca725c65	5	1
06b3eb7c4b11325f4670ee8bf797192c	5	1
05605f5508068d8a724d42360ec1a8f1	5	1
850dea80c7bb496ac3388d2756b784fa	5	1
4025ce7628c913379c32e51ec313c295	5	1
089e857d1123f321ab3912868a9c3de9	5	1
d2549689ff457ae53646f6fc86383d79	5	1
dbe034d7bd68c5191c21d34cc24f66f4	5	1
da60bcf77d2a10e08f2404f3803a12bf	5	1
ef66ec977ae8df1fd6bd734fec4c8117	5	2
4615c33bd0612615823860f432de266a	5	1
0d023ed273812b0193ab0d4dfe0ddf99	5	1
ed4bb998edf68b609e0b58375096ccfd	5	1
6bbc0579297d23452e2625214fcb1eb0	5	1
3ca4389c31d3d83c73460dbc12568704	5	1
9131e973087f8e35822b3f2f24d040e4	4	1
3c0bea88efba1c8f3d84a1916f64300c	4	1
2514c3b40739ea02e9fa0de73867a018	4	1
b3b06dbf37a7eab99bf7ae548c4c0d2b	4	1
087a83bdcc9ba60eaad132de29193a1e	4	2
d8a018f796c5c7043395c6ac1844ab21	4	1
fc38de706763800bc6a93c8e6d373b72	4	1
121e8516a8415e3e0009b2cdedb2dfbe	4	1
5717b96bbe0946e2209b0e14e4f828d3	4	1
43f44d35dcb40b558abed7433cd0d6c1	4	1
f5b1171ba916bb7ff6c0166f590aa85f	4	1
683df4e17f1f1d70012e495bd158a077	4	1
73ecbe31062a71a3019b565882bc8b6c	4	1
c1fa86c28a672d3d1e154fa45acfdb9b	4	1
7ff8313bd679520727ad9818ea14158b	4	1
ca8286e24702cc1b2d29646bccd0f240	4	1
854a549c2385492bcbe225c895e3db57	4	1
63c9ba073dbabfa3f1199a2135ad91ec	4	1
02ff464f2aeb1496df003693b2516c7c	4	2
de39782dafa438932421b40ead1a639b	4	1
805a58a103f2546e9a257a772b163dc1	4	1
4a2206c810686389982b688de795d5a3	4	1
16b4fc34ffc7d658c3003bf71d262527	3	1
194c1f6e6117b461faf8ed089bb45e2f	3	1
094f090854ef4c0813af139198f7fe1c	3	1
fed82b18e87015d480f9f30d646f9761	3	1
76b9f9bd042761ee48a4fa8ac1ede5ca	3	1
5f9b1a144910192fad1bd4a3d8b8b58c	3	1
8b84d4f82dbc4cb715e7a7a6e9e5b846	3	1

9a8fe698727de87bbb2eb1e40e51e4d3	3	1
9c06e714c37f27a159af8b67686d8139	3	1
a318d0193883d429e0199320aa6f4d64	3	1
7cb298480601dafd20b6a5a64ec43368	3	1
797ec82b253d67346dc90592d8817fed	3	1
b45f4bf35843cc2c1c61488fbc51ebf9	3	1
c177aa74e66addffa5ff4e62ba311942	3	1
c21b68031aec4cb9b53987d9bf93babf	3	1
c49a073bdcd7232774f9eb14b7db92e1	3	1
6c2bb40982eab12e7efde4132ca2d1a5	3	1
6a927ff6420d4618b6056b190d28328c	3	1
6944be8af712798697e0a4b34b17d1c6	3	1
c93339eedc52a59a6e584cb3725027b6	3	1
64609bd25f8c73b17c24e0bc6999c18a	3	1
f7833fafd019341311ac8bb613ae47c7	3	1
e577a127861784ad863b076a328025c7	3	1
fc839a87d0c3d233535daf29c989baa6	3	1
eb9a6ff1641504d8b72d679bca7ee599	3	1
370696bb190ade4fb1eaa6929800537e	3	1
3a01392f7a93ee628fee9d7bdf81e328	3	1
4f252794952bf914a2165a589bcf48f9	3	1
3a2c6e082cca7094d01a91ff5f1c4463	3	1
aca79e16df499731584dc67f4fa9f4a7	2	1
0e41e5c44d294b1cc181b68a7289ab08	2	1
fd3f1d44f9767d5abaa084753ceb1f5f	2	1
02225615179dedc38fec61282f96c559	2	1
dbd3787d208606bf0cbe7e74de30a31b	2	1
fb9eb3e4fa2b707587fe404377f55e00	2	1
c733f0e80f8196c1b45924f765cef08b	2	1
c417cde5e4588de1e788a0095a238a89	2	1
ec18fd6241654ee5358da89f6efaa85d	2	1
eb9613cf8dc457220dad25313db1ab53	2	1
062a6977877aacfef233a53cd4ae6a7f	2	1
05f3e6817f0b313564e50a1502e29ff6	2	1
99a685778b5da5c80378ff6d6eb600fd	2	1
99c0ae36ffbf583cf77c1f4c9848c949	2	1
2307bde932214068ba9462f35763404a	2	1
62b3dfe4921ac5d5359ac0af96cbd333	2	1
76bd6b327d8ee5bdc8475077809e7bb7	2	1
353a819a6d6451be3651768c0941a251	2	1
36ac123fed14bdb910433dcd22b9c921	2	1
44191952e54d6de1d65f76674dda8d45	2	1
522348b5dc406bbf180c86ce158259f3	2	1
5290c49da4492a10edd7988865edace6	2	1
56e30419dedfd2d957e6ca571ed36d2e	2	1
58be2b3bf6af851bf0969d74b8bec83a	2	1
75cc0cfb66231e69a8b8867db444a7e9	2	1
99bfaf73cc4b797384e9dabc5f10342a	2	1
8158341cfc498f9b40c942b449dc26bd	2	1
12da7e48fa3887f67f0325ab3bbee90f	2	1

7967684498524f06c6bcdff2c327aa5a	2	1
b71d72336f500d0d08583d03fc52909f	1	1
fd990c15ddaff376ceda14eb94069e3f	1	1
0ea8fddd7b324cf89c2f8b451e341778	1	1
fce3b10b4652e4d04f9bb634c4419ca5	1	1
214e56e9f1178d6f4d1fbbc8631d7fb5	1	1
8f918ef9b5c7347caf6f717c7e9015a9	1	1
3bdc5a64cf58117685d64cc75f890377	1	1
fa742c6f0fe519acd2e64322d000cc94	1	1
f92702d401a3873c6417e7a698b95e4b	1	1
8b1989d79ea5cc5e6aac800b1bc3b3d2	1	1
a1ce8303774e21bdaaa2bc8acfb60f90	1	1
569bcc1c0f806fd9c7f7e47b87c66dc1	1	1
dde7cd6b574df17c17f0f1b6362d3037	1	1
ba6cf4bc54758d2a3ef686d91b6d6a57	1	1
cd80afbadb0797043698ca586260a017	1	1
cc0ab98c0077d46b69172f7d0c335bcd	1	1
cacfc0210433ebc36e55d1cf50bfefc9	1	1
6181fe556c5a503eb2b68d2176cd6d94	1	1
947c443d5fd8a2d721cb383bb070a04a	1	1
c6655e607954b0c5b082f47527a10529	1	1
648a222faa08620fa277a3eaabf6c253	1	1
67aa33d2f3f095e8e1075e6228dd96e6	1	1
7415ff16f1d0d244db13ba32b87ebba0	1	1
b459b4bf78b5a5e203775d99e9302b3d	1	1
76a55a97dfc6db6fd19d5ac820a97495	1	1
78679e4bd376d7e98ea29b6ea7e6b15b	1	1
3436d19e493a19ba6460574920b286e1	1	1

TABLE S9 Table showing the frequencies of ASVs obtained after filtering out ASVs that have less than 2000 frequencies from the merged ASV-table

ASV	Frequency	# of Samples Observed In
986967fd4264ba896aabed4c25f9a4af	1.613.524	112
65496bbf4dc78382fac58935a32b1445	1.608.245	112
598d03e94e39c24ad9210b56ec86c67e	1.162.377	112
1231c484790871f1b02b5b7352494fea	1.137.776	112
5436a782bc7ed40b8ad909655b0af7ce	531.807	112
7db7c174c23363d553779ac968314491	199.405	112
12e264fec25bbe1cc51d233b62ccc963	157.235	81
179cbbc955a119e3c2353f48aa10bc5a	152.983	112
03713a8928ea62b4d6bf82d78a3b15a4	149.974	112
e240646ffd33df766f41a7409825a044	121.117	112
b66fc66e422a26a043680650483f7f8b	84.838	112
3663d41e5a13c3ac52ae7fbe31cfaa6b	68.974	112
4373377f404bb9bf1f5b37c16662cd58	56.862	112
d99f15f84c339c746749b50a1cd1f71d	51.310	112
61a40c5615f3cd435404098a5019af66	51.047	111
97e0d6f1f616009df12e58179ef71fea	50.856	112
af3a07b12e3543eed55870c9056ffaf3	49.499	112
49c110de6638143ac4cc7ae844f93071	42.660	112
b022b31257c230943cf27347f3dfa0c7	41.257	112
1deb11e859c78a082e2e11143f332e38	32.868	112
9e8a870c7a9c57f3d3b734318536d7f0	23.833	92
4541d7f9536c04dd1151a0be0ceee228	23.039	92
ceacdcd5217a7d3be3456a2ce1da6a31	22.150	110
22ca70d16470cad66451b56586898b92	21.063	112
d8f3c828aa33bec6cbd622058dd7e01a	20.359	97
7fb1ca902a4d4a930b91c2492f409968	20.077	112
d51f208ff77a7e9eea303072812164f7	19.837	96
3bf9589bc24ee9c13d3f8e8a819011e4	18.667	64
ac623d76c0e59e6f53aee215a93c8497	13.248	111
58d0e3c2ce468949ea7570310c9cf17c	12.562	110
769cf91f760ccf78dfd1a4e0fbbb0b76	12.050	45
2332e8722d90259636448e1e82d9113e	10.552	71
e5046e5c557d1f90925a8ed986a0ef29	10.293	48
5829f592447e5b0d8784f43150ffb56f	10.268	56
625cea4e18659e393f3dfe3f3db04c3c	10.261	112
05bb66f7bb196677ca067d5c19a424a4	10.066	96
5df82dddb1aa7154e7783d76fbe1ce3d	9.034	109
dd9e28ed02dfba5d507fadcf492d6713	8.743	77
4b347b76b57c2e71705057dd9dd1aed9	8.508	49
e55bb4ef5c583951053e48c2ec554f3f	8.376	103
5a71155f614b32e16173c607d01764a9	8.125	69
83445197c16bee473991239f7e15f11c	8.096	48
68146e68feac64537e12a9dd5a2a7e05	7.607	93
7624e20a982ba894a211695066600830	6.234	89
a2d76b570fa0469135640884599a2af2	6.138	106
1c9d49371cb8032609739d5de05f50e5	5.901	34
05a630bb34d7f103ca53692a43139459	5.536	34
33cd3dcab2602b1bb11a77c58850e841	5.393	107

fcb419099fe77ad65f097cb4bc49a4a7	5.326	52
831a520fbfce915b3d2081449e9aa0a7	4.746	71
ad757d7046bd853d89f142d2c8466637	4.484	112
481abe1c240a27677005da31d0d7a6c0	4.465	105
91d20ce368b224958b151ff03440ef98	3.636	57
6c55ab68cd43b9974908c956a8a6ac9d	3.631	97
9ed27f4bddcb1afb25681e918566a55d	3.409	20
01591a6731e83bd7b04c607d8b6632eb	2.964	40
77ac02c2d6e75ac848fd96f113337ca0	2.548	39
cc231c20ce7de17921d5e22f37ee0fcc	2.507	103
6d23cba5806cb0cd964a510619774732	2.311	28
9b028b89b8392d8f3d09fe92fb8f2b98	2.167	17
08992aedd5f963ec4c51688e4d980de5	2.036	4

TABLE S10 Linear mixed-effects model results for *Tv sulfidiphilus* relative abundances in oxic reactors for all six experimental runs*

Variable or parameter	Estimate	SE	Z-score	P value
Intercept	-0,103	0,213	-0,483	0,629
Duration_of_operation	0,02	0,01	2,097	0,036
HRT	0,003	0,013	0,227	0,821
Duration_of_operation:HRT	-0,001	0,001	-1,549	0,121
Sulfide	0,307	0,494	0,622	0,534
Duration_of_operation:Sulfide	-0,028	0,019	-1,463	0,143
HRT:Sulfide	0,012	0,042	0,277	0,782
Duration_of_operation:HRT:Sulfide	0,002	0,002	1,234	0,217

*Parameter estimate (coefficient), standard error, Z score, and P value for each model parameter.

TABLE S11 Linear mixed-effects model results for *A. ehrlichii* relative abundances in oxic reactors for all six experimental runs**

Variable or parameter	Estimate	SE	Z-score	P value
Intercept	0,393	0,141	2,792	0,005
Duration_of_operation	-0,015	0,007	-2,105	0,035
HRT	-0,005	0,009	-0,637	0,524
Duration_of_operation:HRT	0,001	0	1,857	0,063
Sulfide	-0,24	0,324	-0,74	0,46
Duration_of_operation:Sulfide	0,024	0,014	1,646	0,1
HRT:Sulfide	0,006	0,028	0,206	0,837
Duration_of_operation:HRT:Sulfide	-0,002	0,001	-1,328	0,184

**Parameter estimate (coefficient), standard error, Z score, and P value for each model parameter.

REFERENCES

1. de Rink R, Gupta S, Piccioli de Carolis F, Liu D, ter Heijne A, Klok JBM, Buisman CJN. 2021. Effect of process conditions on the performance of a dual-reactor biodesulfurization process. J Environ Chem Eng 9:106450.
